# Early senescence and production of senescence-associated cytokines are major determinants of radioresistance in head-and-neck squamous cell carcinoma

**DOI:** 10.1038/s41419-021-04454-5

**Published:** 2021-12-15

**Authors:** Ulrike Schoetz, Diana Klein, Julia Hess, Seyd Shnayien, Steffen Spoerl, Michael Orth, Samet Mutlu, Roman Hennel, Anja Sieber, Ute Ganswindt, Benedikt Luka, Andreas R. Thomsen, Kristian Unger, Verena Jendrossek, Horst Zitzelsberger, Nils Blüthgen, Claus Belka, Steffen Unkel, Bertram Klinger, Kirsten Lauber

**Affiliations:** 1 Department of Radiation Oncology, University Hospital, LMU München, Munich, Germany; 2 Department of Radiotherapy and Radiooncology, Philipps-University Marburg, University Hospital Gießen and Marburg, Marburg, Germany; 3 Institute of Cell Biology (Cancer Research), University of Duisburg-Essen, University Hospital, Essen, Germany; 4 Research Unit Radiation Cytogenetics, Helmholtz Center Munich, German Research Center for Environmental Health GmbH, Neuherberg, Germany; 5 Clinical Cooperation Group ‘Personalized Radiotherapy in Head and Neck Cancer’ Helmholtz Center Munich, German Research Center for Environmental Health GmbH, Neuherberg, Germany; 6 German Cancer Consortium (DKTK), Partner site Munich, Munich, Germany; 7 German Cancer Research Center (DKFZ), Heidelberg, Germany; 8 Institute of Pathology, Charite-Universitätsmedizin Berlin, Berlin, Germany; 9 IRI Life Sciences, Humboldt University of Berlin, Berlin, Germany; 10 Department of Therapeutic Radiology and Oncology, Medical University of Innsbruck, Innsbruck, Austria; 11 Division for Cariology, Department of Operative Dentistry and Periodontology, Center for Dental Medicine, Medical Center - University of Freiburg, Freiburg im Breisgau, Germany; 12 Department of Radiation Oncology, Medical Center – University of Freiburg, Freiburg im Breisgau, Germany; 13 German Cancer Consortium (DKTK), Partner site Freiburg, Freiburg im Breisgau, Germany; 14 German Cancer Consortium (DKTK), Partner Site Berlin, Berlin, Germany; 15 Department of Medical Statistics, University Medical Center Goettingen, Goettingen, Germany

**Keywords:** Radiotherapy, Head and neck cancer, Senescence, Tumour heterogeneity

## Abstract

Resistance against radio(chemo)therapy-induced cell death is a major determinant of oncological treatment failure and remains a perpetual clinical challenge. The underlying mechanisms are manifold and demand for comprehensive, cancer entity- and subtype-specific examination. In the present study, resistance against radiotherapy was systematically assessed in a panel of human head-and-neck squamous cell carcinoma (HNSCC) cell lines and xenotransplants derived thereof with the overarching aim to extract master regulators and potential candidates for mechanism-based pharmacological targeting. Clonogenic survival data were integrated with molecular and functional data on DNA damage repair and different cell fate decisions. A positive correlation between radioresistance and early induction of HNSCC cell senescence accompanied by NF-κB-dependent production of distinct senescence-associated cytokines, particularly ligands of the CXCR2 chemokine receptor, was identified. Time-lapse microscopy and medium transfer experiments disclosed the non-cell autonomous, paracrine nature of these mechanisms, and pharmacological interference with senescence-associated cytokine production by the NF-κB inhibitor metformin significantly improved radiotherapeutic performance in vitro and in vivo. With regard to clinical relevance, retrospective analyses of TCGA HNSCC data and an in-house HNSCC cohort revealed that elevated expression of CXCR2 and/or its ligands are associated with impaired treatment outcome. Collectively, our study identifies radiation-induced tumor cell senescence and the NF-κB-dependent production of distinct senescence-associated cytokines as critical drivers of radioresistance in HNSCC whose therapeutic targeting in the context of multi-modality treatment approaches should be further examined and may be of particular interest for the subgroup of patients with elevated expression of the CXCR2/ligand axis.

## Graphical abstract

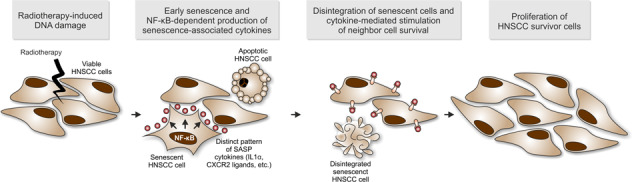

## Introduction

Induction of tumor cell death and the abrogation of clonogenic survival represent primary objectives of anti-cancer radio(chemo)therapy. However, established tumors commonly exhibit multiple mechanisms of resistance which undermine therapeutic approaches and thus drive treatment failure. Defects in the regulation of cell death modalities, hyperactivated survival signaling, amplified detoxification of xenobiotics and/or reactive oxygen species, and accelerated DNA repair are commonly observed [1]. Notably, no “one-fits-all” mechanism can be denominated, and the evolution of precision medicine demands in-depth analyses of therapy resistance in distinct cancer entities.

In this regard, the role of therapy-induced senescence is of specific interest. Initially described as an irreversible form of cell-cycle arrest and — in principle — a desirable endpoint of anti-cancer therapy, tumor cell senescence has meanwhile been recognized to be an ambiguous player in cancer biology [2]. Whereas its crucial role in therapy-induced tumor control is well-established [3], accumulating evidence suggests a relevant contribution of cellular senescence to tumor progression, metastasis formation, and therapy resistance — in cell autonomous as well as non-cell autonomous ways [4, 5]. Accordingly, the reversibility of senescent cell cycle arrest and the impact of the senescence-associated secretory phenotype (SASP) on tumor cell survival, stemness, proliferation, and dissemination moved into the focus of scientific interest [6, 7].

Here, we systematically analyzed resistance against radiotherapy in a panel of human head-and-neck squamous cell carcinoma (HNSCC) cell lines in vitro and in vivo. We integrated clonogenic survival data with molecular and functional data on DNA damage repair, induction of apoptosis and senescence, as well as SASP cytokine production. A positive correlation between radioresistance and early senescence induction accompanied by NF-κB-dependent production of distinct SASP cytokines — most importantly ligands of the chemokine receptor CXCR2 — was observed. Time-lapse microscopy and medium transfer experiments revealed that this axis operated in a non-cell autonomous, paracrine manner, and pharmacological interference with SASP cytokine production by the NF-κB inhibitor metformin significantly improved radiotherapeutic efficacy in vitro and in vivo. Retrospective analyses of the radio(chemo)therapy TCGA HNSCC subcohort and an in-house radio(chemo)therapy HNSCC cohort showed that elevated expression of CXCR2 and/or its ligands associates with impaired treatment outcome. Accordingly, our results suggest that SASP targeting combined modality radiotherapy — preferentially with agents that have been in clinical use for years, such as metformin — represents a promising and timely to implement strategy for radioresistant HNSCC.

## Results

Resistance against radiation-induced cell death is a major challenge for the treatment of HNSCC and a crucial driver of radiotherapeutic treatment failure. The underlying mechanisms remain poorly defined, and apart from the human papillomavirus (HPV) status, no molecular prognosticators have clinically yet been implemented. The present study was designed to identify key players of radiation-induced cell death resistance and clonogenic survival in HNSCC by integrating different levels of cell biological and molecular data from a panel of seven human HNSCC cell lines (Supplementary Table 1).

### Clonogenic survival in vitro and xenograft growth in vivo differ strongly across a panel of human HNSCC cell lines upon radiotherapy

**Fig. 1:**
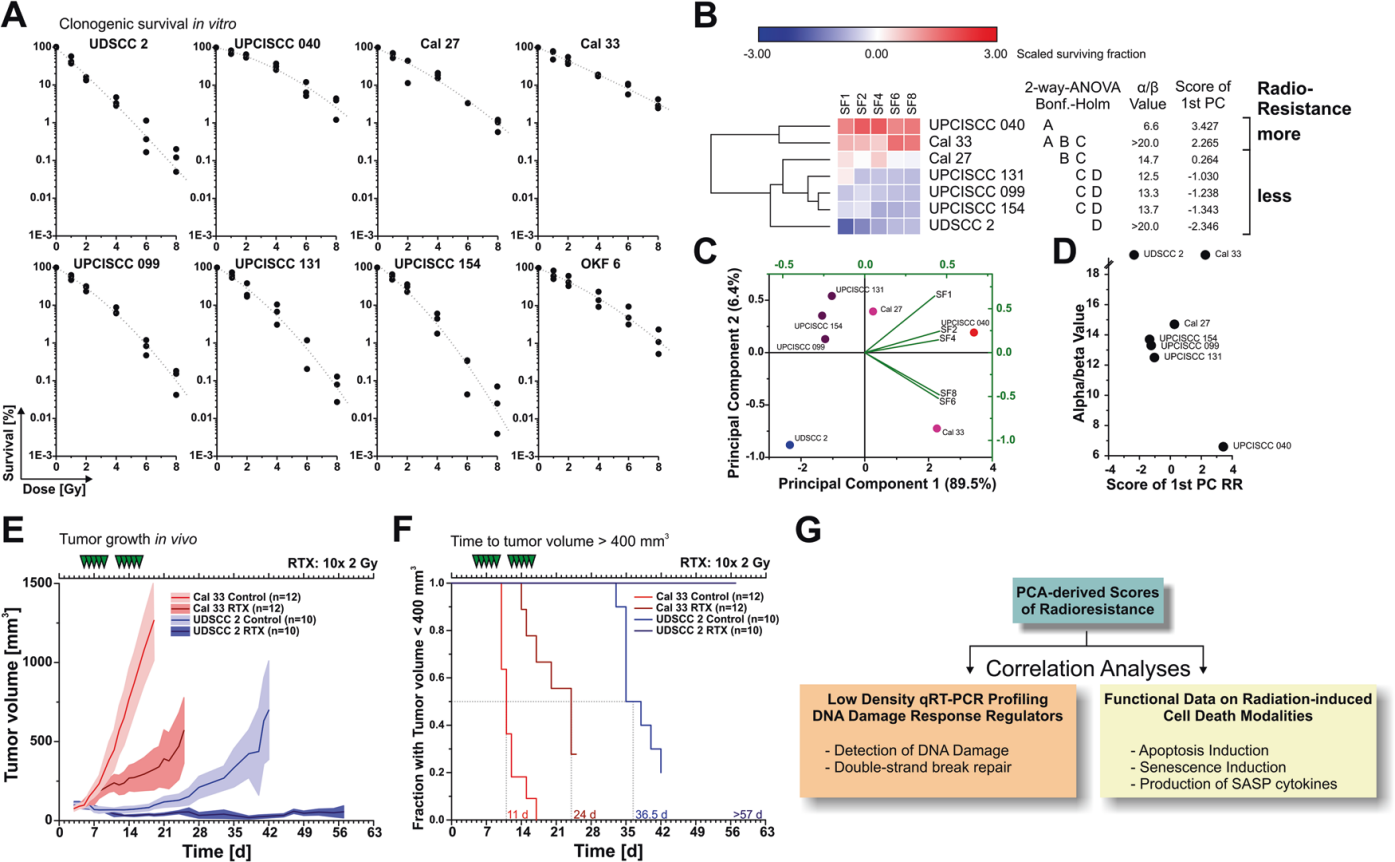
Clonogenic survival in vitro and xenograft growth in vivo differ strongly across a panel of human HNSCC cell lines upon radiotherapy. **A** Clonogenic survival of 7 HNSCC cell lines and human hTERT-immortalized OKF 6 keratinocytes as measured by colony formation assays upon irradiation at 0–8 Gy in vitro. Results of three independent experiments are depicted with linear-quadratic fitting functions superimposed. **B** Unsupervised hierarchical clustering of z-transformed survival data from **A** (without OKF 6 data). α/β-values and the scores of the 1st principal component (PC) as extracted *via* PCA are indicated. Capital letters depict grouping results obtained by two-way ANOVA with means comparison according to Bonferroni-Holm. Results from cell lines which do not share a letter are statistically significantly different. **C** PCA-derived biplot of z-transformed clonogenic survival data from **A** (without OKF 6 data). **D** Graph illustrating the lacking correlation between α/β-values and the scores of the 1st PC as markers of radioresistance (RR). **E** Growth curves of heterotopic xenograft tumors on NMRI nude mice ± fractionated irradiation with 10 × 2 Gy as measured for two representative cell lines with high and low in vitro radioresistance. Means with upper and lower 95% confidence intervals are shown. **F** Time to tumor volume > 400 mm^3^ analyses of growth curves shown in **E**. **G** Schematic workflow of subsequent correlation analyses with PCA-derived scores of radioresistance.

First, clonogenic survival of all cell lines was examined upon radiation at 0–8 Gy in vitro. Clear differences were observed among the cell line panel with UDSCC 2 and UPCISCC 154, the two HPV-positive cell lines, being the most sensitive ones, and UPCISCC 040 and Cal 33 being the most resistant ones — more resistant than hTERT-immortalized oral human OKF 6 keratinocytes (Fig. 1A, B). These differences were exemplarily confirmed in vivo when xenotransplants of selected cell lines were treated with clinically relevant fractionation schedules of daily doses of 2 Gy (Fig. 1E, F). Quantitative measures of radioresistance were obtained by dimensionality reduction *via* principal component analysis (PCA) of the z-transformed clonogenic survival dataset (Fig. 1B–D). As shown previously, PC1 represented a homogenously loaded and informative measure of radioresistance and was only in part related to the historical α/β-value (Fig. 1B–D) [8]. In order to identify central determinants of radioresistance, we next performed correlation analyses of the radioresistance scores with different cell biological characteristics, including DNA damage response regulator expression, radiation-induced apoptosis and senescence, as well as production of senescence-associated cytokines (Fig. 1G).

### mRNA expression analysis of DNA double-strand break repair regulators identifies PARP2 as a potential candidate for targeted radiosensitization of distinct HNSCC cell lines

Introduction of lethal chromosomal damage (in the case of photon radiation mainly DNA double-strand breaks) is considered to be the major cause of radiation-induced cell death. Accordingly, differences in the abundance and/or the functionality of proteins involved in DNA damage repair mechanisms can lead to more or less radioresistant phenotypes [9–12]. We utilized low-density qRT-PCR profiling to examine the mRNA expression levels of key regulators of DNA double-strand break repair (Supplementary Fig. 1A). Unsupervised hierarchical clustering and PCA of the mRNA expression data separated the cell line panel into two main clusters: UPCISCC 040 and Cal 27 cells (both HPV-negative and rather radioresistant) vs. a second cluster comprising an HPV-negative and an HPV-positive subcluster (Supplementary Fig. 1A, B). These findings are in line with previous reports showing that HPV-positive cell lines show a higher degree of radiosensitivity due to impaired DNA double-strand break repair [13]. Moreover, these results encourage therapy concepts with definitive radiotherapy (without prior surgery) for HPV-positive pharyngeal HNSCC where surgery may be extremely amputating [14].

The most obvious cluster separating input variable was expression of PARP2 (Supplementary Fig. 1C). Although PARP2 expression was strongly upregulated in all HNSCC tumor cell lines as compared to hTERT-immortalized OKF 6 cells, particularly high levels were observed in UPCISCC 040 and Cal 27 cells. In these two cell lines, PARP2 was specifically or even uniquely (in the case of Cal 27) overexpressed in comparison to all other DNA damage response regulators analyzed (Supplementary Fig. 1A, C), suggesting that it may represent a distinct therapeutic vulnerability. In consequence, pharmacological inhibition of PARP1/2 by olaparib significantly reduced clonogenic survival of UPCISCC 040 and Cal 27 cells upon radiotherapy, whereas survival of OKF 6 cells remained largely unaffected (Supplementary Fig. 1D). Similarly, silencing of PARP2 expression by transfection of UPCISCC 040 cells with a PARP2-specific siRNA oligonucleotide led to a significant decrease in clonogenic survival upon irradiation, yet to a slightly lesser extent (Supplementary Fig. 1E, F). These results identify PARP2 as promising vulnerability of targeted radiosensitization for PARP2 high expressing HPV-negative HNSCC [15]. Nevertheless, the correlation between PARP2 expression and overall radioresistance as determined by the radioresistance scores (Fig. 1B) was rather weak (Spearman’s ρ = 0.34), and no convincing positive correlation was observed for any other of the DNA double-strand break repair regulators analyzed.

### Induction of apoptosis negatively correlates with radioresistance, but inhibition of apoptosis by the poly-caspase inhibitor zVAD-fmk has no significant effect on clonogenic survival upon irradiation in vitro

The major consequences of unrepaired and/or lethal DNA damage comprise transit into aberrant mitosis, or induction of cellular senescence, respectively, depending on the extent of DNA damage and the functionality of cell cycle checkpoints [16, 17]. Upon photon irradiation at doses used in the present study, tumor cells with defective cell cycle checkpoints typically re-enter the cell cycle and resume proliferation after a lag time of attempted DNA repair. However, if the load of unrepaired and/or lethal DNA damage is too high, this will lead to mitotic catastrophe ultimately culminating in cell death. We, therefore, measured induction of apoptosis in the HNSCC cell line panel upon irradiation at 0–8 Gy over 8 days. Strongly varying responses were observed, although radiation doses ≥ 4 Gy collectively stimulated a well-detectable apoptotic response in the majority of cell lines at day 4 after irradiation and later (Supplementary Fig. 2A). In order to find similarities between the cell lines and to integrate this dataset with the radioresistance scores, the apoptosis data were z-transformed and subjected to hierarchical clustering and PCA (Supplementary Fig. 2B, C). Hierarchical clustering separated the weakly apoptosing, rather radioresistant cell lines Cal 33 and Cal 27 from all others, and the PCA-derived apoptosis scores revealed a significant negative correlation with the radioresistance scores (Supplementary Fig. 2C). Functionally, however, apoptosis inhibition by adding the poly-caspase inhibitor zVAD-fmk did not affect clonogenic survival upon irradiation (Supplementary Fig. 2D), although the effectivity of zVAD-fmk treatment was confirmed by caspase activity measurements in whole-cell lysates (Supplementary Fig. 2E). This was observed for several cell lines, irrespective of their apoptosis or radioresistance levels, suggesting that induction of apoptosis is dispensable for clonogenic inactivation in response to ionizing radiation and/or those other modes of cell death may contribute as well — at least in our in vitro setup.

### Ionizing irradiation also stimulates necrotic cell death, but induction of necrosis does not correlate with radioresistance in vitro

We next analyzed the induction of necrosis in the HNSCC cell line panel upon irradiation at 0–8 Gy over 8 days. In the majority of cell lines, the necrotic response was stronger than the apoptotic response observed, particularly at doses ≥ 4 Gy. Furthermore, we noticed relevant differences in terms of necrosis kinetics across the cell line panel. For UPCISCC 040, Cal 33, UPCISCC 099, and UPCISCC 131 cells induction of necrosis appeared to parallel and follow the induction of apoptosis, suggesting that post-apoptotic, secondary necrosis was the dominant mechanism. In contrast, for the other cell lines induction of necrosis was observed with much faster kinetics, higher amplitudes, and seemingly independent of apoptosis induction. Dimensionality reduction, hierarchical clustering, and PCA of the necrosis dataset in analogy to the workflow used for the apoptosis data did not reveal any quantitative association and/or correlation of necrosis induction upon radiotherapy with radioresistance. However, the three most radioresistant cell lines UPCISCC 040, Cal 33, and Cal 27 showed the fastest recovery kinetics beginning after day 4–6 upon irradiation (Supplementary Fig. 3).

### Radiation-induced senescence positively correlates with radioresistance, and senescent cell-derived soluble factors support clonogenic survival in vitro

**Fig. 2:**
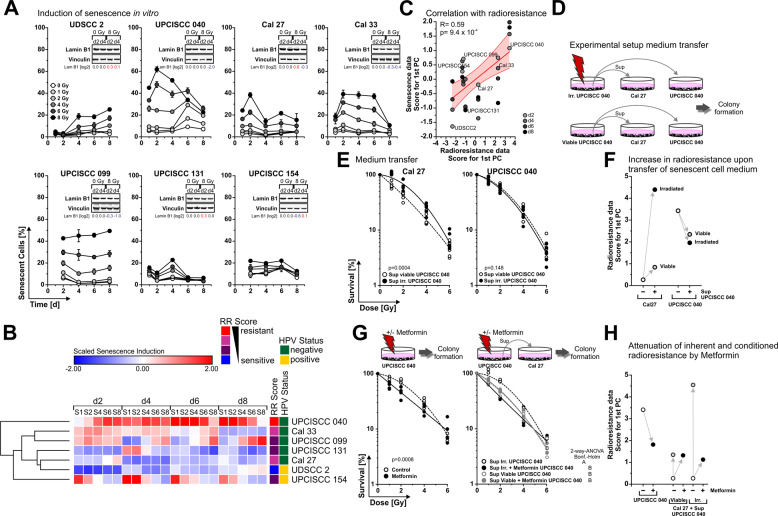
Radiation-induced senescence positively correlates with radioresistance, and senescent cell-derived soluble factors support clonogenic survival in vitro. **A** Induction of cellular senescence upon irradiation at 0–8 Gy as measured after 1–8 d by flow cytometry (gating strategy shown in Supplementary Fig. 4A). Means ± SD of triplicates are depicted. Inserts show Lamin B1 protein expression levels as detected and quantified by western blot analyses with vinculin as a loading control. **B** Unsupervised hierarchical clustering of z-normalized senescence data from **A**. **C** Correlation analysis of the scores of the 1st PC of radioresistance (Fig. 1C) with the scores of the 1st PC of senescence induction at different time points (day 2–8) with superimposed linear regression ± upper and lower 95% confidence intervals. Pearson’s R and *p*-value are indicated. **D** Sketch depicting the experimental setup of medium transfer experiments performed in **E**. **E** Colony formation after medium transfer. Cal 27 and UPCISCC 040 cells were irradiated at 0–6 Gy, and colony formation was allowed in the presence of conditioned supernatants of UPCISCC 040 cells. Results of five independent experiments are shown with linear-quadratic regression superimposed. *p*-values were calculated by two-way ANOVA. **F** Survival data from **E** was z-transformed and projected to PC1 from Fig. 1B with the respective loadings, and the resulting scores of PC1 are shown in comparison to the scores of the PC1 from Fig. 1C. Arrows indicate the trend of increasing/decreasing radioresistance. **G** Colony formation of UPCISCC 040 and Cal 27 cells as measured upon irradiation at 0–6 Gy in the presence of metformin, or conditioned medium generated in the presence of metformin. Results of four (left panel) or three (right panel) independent experiments are shown with linear-quadratic regression superimposed. *p*-values were calculated by two-way ANOVA. Capital letters depict grouping results obtained by two-way ANOVA with means comparison according to Bonferroni-Holm. Results from datasets that do not share a letter are statistically significantly different. **H** Survival data from **G** was z-transformed and projected to PC1 from Fig. 1B with the respective loadings, and the resulting scores of PC1 are shown in comparison to the scores of PC1 from Fig. 1C. Arrows indicate the trend of increasing/decreasing radioresistance.

In cells with operational cell cycle checkpoints, mitotic catastrophe upon overwhelming DNA damage is prevented by induction of cellular senescence, a long-term and largely irreversible cell cycle arrest with distinct phenotypic alterations, and a complex secretome [17, 18]. However, senescence has been described to have ambiguous effects on tumor progression and tumor cell repopulation during anti-cancer therapy [4, 5]. So, we examined the induction of senescence in the HNSCC cell line panel upon radiation at 0–8 Gy over 8 days (Supplementary Fig. 4A, B). Whereas the levels of senescence induction generally increased with radiation dose, the overall patterns and dynamics were rather heterogeneous (Fig. 2A, B): UPCISCC 040, Cal 27, and Cal 33 cells, the three most radioresistant cell lines, showed an early and strong induction of senescence, peaking at day 2 after irradiation and declining afterward by the disintegration of senescent cells and repopulation of viable cells (Supplementary movie, Supplementary Fig. 4C). In contrast, the more sensitive cell lines exhibited a weaker senescence response with delayed onset (UDSCC 2), prolonged duration (UPCISCC 099), or very low overall amplitude (UPCISCC 154 and UPCISCC 131), respectively. Analogous to the workflow applied to the apoptosis dataset, the senescence dataset was subjected to dimensionality reduction *via* PCA. Intriguingly, the extracted senescence scores revealed a significant positive correlation with the radioresistance scores (Fig. 2C). Although at first sight counter-intuitive, similar findings have already been made by others. As such, senescence has been reported to promote clonogenic survival by involving cell autonomous (formerly senescent cells which resume proliferation) as well as non-cell autonomous mechanisms (senescent cells which favor proliferation of neighboring cells by release of SASP cytokines) [6, 19]. The former appears to be largely limited to experimental model systems with genetically switchable regulators of senescence, and our time-lapse movies did not provide evidence for senescent cells re-entering proliferation (Supplementary movie). We, therefore, elucidated if senescent cells can support proliferation of neighboring cells *via* released SASP factors in medium transfer experiments. Conditioned medium of irradiated, senescent UPCISCC 040 cells significantly augmented clonogenic survival of Cal 27 cells as compared to conditioned medium of viable, non-senescent UPCISCC 040 cells (Fig. 2E). Quantitatively, the transfer of conditioned medium from senescent UPCISCC 040 increased the radioresistance score of Cal 27 to the level of UPCISCC 040 cells (Fig. 2F). This was not observed in the case of homotypic medium transfer from UPCISCC 040 producer to UPCISCC 040 recipient cells, presumably because UPCISCC 040 recipient cells produced sufficient survival SASP cytokines by themselves. Vice versa, when SASP cytokine production in UPCISCC 040 cells was inhibited by adding the NF-κB inhibitor metformin [20], clonogenic survival of UPCISCC 040 cells upon irradiation was significantly reduced, and the increase in radioresistance of Cal 27 cells by transfer of conditioned medium from senescent but not from viable UPCISCC 040 cells was essentially abrogated (Fig. 2G, H). Taken together, the cell fate data are shown in our study allow the following conclusions: Clonogenic survival of HNSCC cells upon irradiation is driven by a small subpopulation of cells (less than 10% at irradiation doses ≥ 4 Gy). This can be strongly supported by senescent cell-derived soluble factors which are produced in an NF-κB-dependent manner early upon irradiation (as is the case for radioresistant UPCISCC 040 and Cal 33 cells). But clonogenic survival is not influenced by the degree of apoptosis and/or necrosis induction in the rest of the cell population — at least in the dose range and the model systems employed in our study.

### The pattern of SASP cytokine production upon radiotherapy is different between radioresistant and radiosensitive HNSCC xenotransplants in vivo and normal oral human keratinocytes ex vivo

**Fig. 3:**
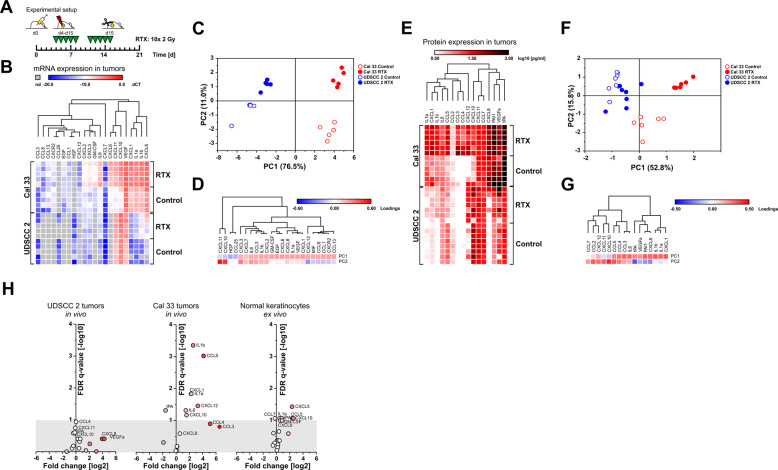
The pattern of SASP cytokine production upon radiotherapy is different between radioresistant and radiosensitive HNSCC xenotransplants in vivo and normal oral human keratinocytes ex vivo. **A** Schematic of experimental setup for xenograft treatment and tissue sampling. Cytokine production in xenograft explants was assessed by qRT-PCR analyses (**B**–**D**) and multiplex ELISAs (**E**–**G**). **B** Unsupervised hierarchical clustering of relative mRNA expression levels (dCT-values normalized on a reference gene matrix of 18 S rRNA, β2-Microglobulin, and δ-ALAS). nd indicates not detectable. *n* = 5 animals for all groups. **C** PCA of data shown in **B** plotted in the space of PC1 and PC2. PC1 separates cell lines, PC2 separates irradiated vs. non-irradiated samples. **D** Unsupervised hierarchical clustering of the loadings of the input variables on PC1 and PC2. **E** Cytokine production in Cal 33 and UDSCC 2 xenografts as measured by multiplex ELISAs. Unsupervised hierarchical clustering with *n* = 6 for Cal 33 control and RTX, *n* = 8 for UDSCC 2 control, and *n* = 7 for UDSCC 2 RTX. **F** PCA of the cytokine release data from **E**. PC1 separates cell lines, PC2 separates irradiated vs. non-irradiated samples only in case of Cal 33. **G** Unsupervised hierarchical clustering of the loadings of the input variables on PC1 and PC2. **H** Cytokines released by normal human oral keratinocytes of *n* = 3 healthy donors on day 4 ± irradiation at 4 Gy in comparison to cytokines detected in irradiated UDSCC 2 and Cal 33 tumors from **E**.

It is becoming increasingly clear that acute and chronic senescence are characterized by distinct SASP factors with diverging consequences for tissue regeneration and tumor progression [21–23]. SASP cytokines of acute senescence whose production is orchestrated *via* the inflammasome/IL1α/NF-kB axis are considered to exert largely pro-tumorigenic functions [5, 24, 25], whereas interferon-dependent SASP factors of chronic senescence have been reported to be involved in degenerative processes and normal tissue complications upon chemo- and/or radiotherapy [2, 26]. We, therefore, analyzed the pattern of SASP cytokine production in exemplary xenotransplants of radiosensitive and radioresistant HNSCC cell lines in vivo (Fig. 3A). qRT-PCR analyses revealed obvious differences in basal and radiation-induced cytokine expression of radioresistant Cal 33 and radiosensitive UDSCC 2 xenotransplants with elevated expression levels of a wider spectrum of cytokines being observed in Cal 33 tumors (Fig. 3B). PCA of this dataset disclosed that PC1 and PC2 separated the samples according to the cell line (PC1) and the treatment (PC2) (Fig. 3C, D). Irradiated Cal 33 tumors exhibited a broad range induction of diverse acute SASP cytokines, including IL1α, IL1β, CXCL1, CXCL2, CXCL3, CXCL6, CXCL8, GM-CSF, and IL6. In contrast, UDSCC 2 tumors showed a much more confined spectrum of SASP cytokines dominated by the interferon targets CXCL10 and CXCL11 and a clearly weaker induction of acute SASP cytokines upon irradiation as compared to Cal 33 tumors (Fig. 3B, C). With a slightly smaller panel of cytokines, similar findings were obtained on the protein level by multiplex ELISA measurements (Fig. 3E–H), thus confirming that the pattern and the amplitude of SASP cytokine production upon radiotherapy are different between radioresistant Cal 33 and radiosensitive UDSCC 2 tumors.

The spectrum of SASP cytokines upregulated in irradiated Cal 33 tumors was also overtly different from the one produced in irradiated ex vivo cultures of normal human oral keratinocytes. The general amplitude of cytokine induction in normal keratinocytes was not as strong, and CCL5 and CXCL10 were the only cytokines with significant upregulation (FDR *q*-value < 0.1) that were also found to be upregulated in irradiated Cal 33 tumors (Fig. 3H). These results point towards a distinct and cell type-specific regulation of SASP cytokines in radioresistant HNSCC cells as compared to radiosensitive HNSCC cells and normal oral keratinocytes.

### Time course analyses of SASP cytokine mRNA production in HNSCC cell lines in vitro reveal a positive correlation between CXCR2 ligand induction upon irradiation and radioresistance

**Fig. 4:**
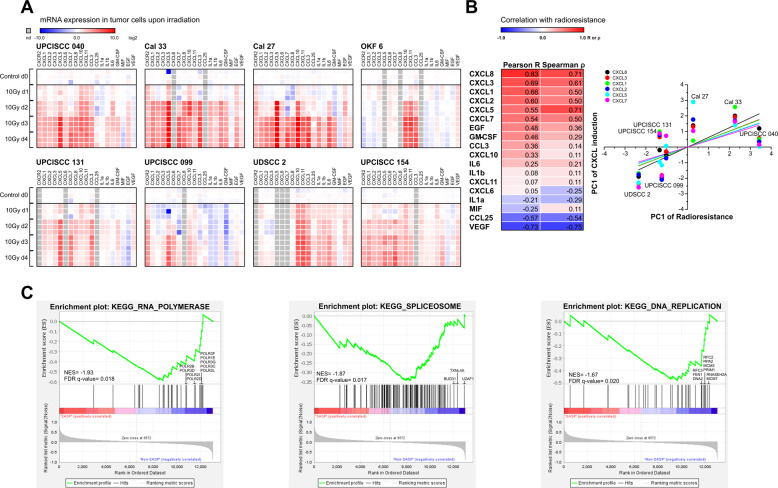
Time course analysis of cytokine mRNA production in HNSCC cell lines upon irradiation in vitro reveals a positive correlation between CXCR2 ligand induction and radioresistance, and a negative enrichment in gene sets associated with RNA transcription, RNA splicing, and DNA replication. **A** Heat map of cytokine mRNA expression levels in HNSCC cell lines on day 0–4 after irradiation at 10 Gy. Results of 3 replicates per time point as determined by the ddCT-method with a reference gene matrix of 18 S rRNA, β2-Microglobulin, and δ-ALAS are shown. **B** Correlation analyses of cytokine induction and radioresistance (without OKF 6 data). Cytokine expression data shown in **A** (without OKF 6 data) were subjected to PCA, and the resulting scores of the 1st PC were used for correlation analyses (parametric and non-parametric) with the scores of the 1st PC of radioresistance as determined in Fig. 1. The table is sorted in descending order of Pearson’s R, and the graph shows the correlation results of CXCR2 ligands (CXCL1, CXCL2, CXCL3, CXCL5, CXCL7, and CXCL8). **C** Gene set enrichment analysis of UPCISCC 040, Cal 33, and Cal 27 cells vs. all others (KEGG subset of MSigDB canonical pathways). Significantly enriched gene sets with FDR *q*-values < 0.1 are shown.

Next, we examined if the induction of distinct SASP cytokines is associated with the degree of radioresistance. To this end, time course qRT-PCR studies of SASP cytokines were performed in the HNSCC cell line panel. Cytokine induction was strongest in the three most radioresistant cell lines UPCISCC 040, Cal 33, and Cal 27. When PCA-derived scores of induction for each cytokine were subjected to correlation analyses with the radioresistance scores, the top hits interestingly comprised the ligands of the chemokine receptor CXCR2 (CXCL1–3, CXCL5, CXCL7, and CXCL8 (Fig. 4A, B)) and the CXCR2 receptor itself. Gene set enrichment analyses (GSEA) of RNA sequencing data from all cell lines (without further treatment) revealed a selective negative enrichment of gene sets involved in RNA transcription, RNA splicing, and DNA replication in the three most radioresistant cell lines (Fig. 4C). This may be of relevance for cell type-specific SASP factor production, because several members of these gene sets, such as U2AF1, have been described to play pivotal roles in intron retention and alternative splicing mechanisms during SASP cytokine production, including CXCL8 [27, 28].

### SASP cytokine production upon irradiation can be inhibited by metformin and translates into improved therapeutic efficacy of radiotherapy in vivo

**Fig. 5:**
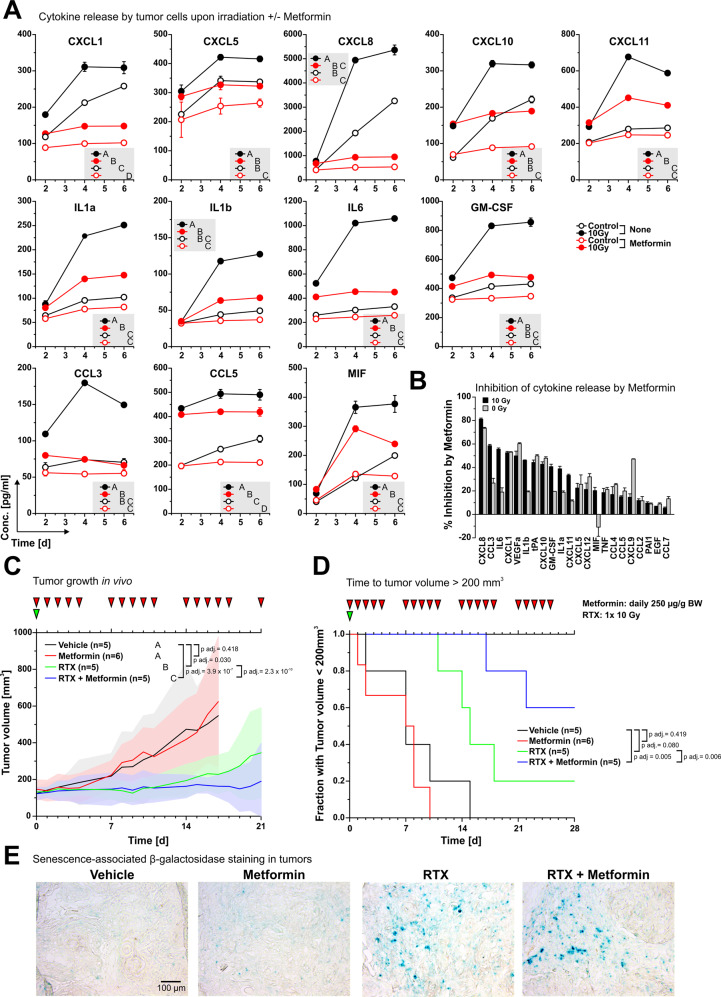
SASP cytokine production upon irradiation can be inhibited by metformin and translates into improved therapeutic efficacy of radiotherapy in vivo. **A** Cytokine protein levels in supernatants of UPCISCC 040 cells on day 0–6 after irradiation at 0 Gy or 10 Gy + /− 5 mM metformin as determined by multiplex ELISAs. Means ± SD of triplicates are shown. Capital letters depict grouping results obtained by 2-way ANOVA with means comparison according to Bonferroni-Holm. Treatments which do not share a letter are statistically significantly different. **B** Inhibition of cytokine production by metformin as normalized to vehicle controls in 0 Gy and 10 Gy samples on day 4 presented in descending order depending on the extent of inhibition (means + SD). **C** Growth curves of heterotopic UPCISCC 040 xenografts on NMRI nude mice + /- irradiation with 1×10 Gy ± week daily doses of 250 µg/g body weight metformin. Means with upper and lower 95% confidence intervals are shown. *p*-values were calculated by two-way ANOVA and subjected to *post-hoc* Bonferroni-Holm correction. **D** Time to tumor volume > 200 mm^3^ analyses of growth curves shown in **D**. *p*-values were determined by log-rank testing with subsequent Bonferroni-Holm correction. **E** Detection of senescent cells by immunohistochemical staining of senescence-associated β-gal in cryosections of explanted tumors (day 5 after therapy start).

Our results so far have shown that the induction of senescence and the production of a distinct acute SASP cytokine pattern with enrichment of CXCR2 ligands upon irradiation are associated with radioresistance in models of HNSCC. Since NF-κB is a master regulator of acute SASP cytokine production [20] and our clonogenic survival experiments have shown that inhibition of NF-κB by metformin interferes with inherent as well as conditioned radioresistance in our HNSCC models (Fig. 2G), we next analyzed cytokine production by radioresistant UPCISCC 040 cells upon ionizing irradiation in the presence or absence of metformin. A significant inhibitory effect was observed for the majority of cytokines examined — particularly for but not limited to the CXCR2 ligands CXCL1, CXCL5, and CXCL8 (Fig. 5A, B). In vivo, combination of a single dose radiotherapy regimen (1 × 10 Gy) with administration of metformin significantly improved the treatment outcome in UPCISCC 040 xenotransplants with regard to tumor growth and time to tumor volume > 200 mm^3^ (Fig. 5C, D). Addition of metformin did not noticeably inhibit radiotherapy-induced tumor cell senescence in vivo (Fig. 5E), allowing the conclusion that metformin-mediated interference with SASP cytokine production, not inhibition of senescence induction per se enhances radiotherapy-induced tumor growth control in HNSCC xenotransplants.

### Metformin improves the outcome of fractionated radiotherapy only in the subgroup of tumors with attenuated cytokine response

**Fig. 6:**
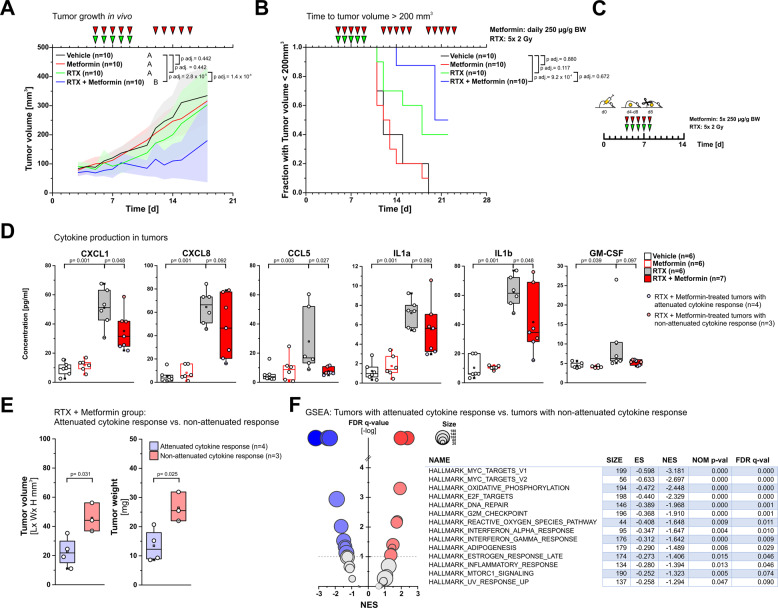
Metformin improves the outcome of fractionated radiotherapy only in the subgroup of tumors with attenuated cytokine response. Cal 33 cells were heterotopically transplanted onto NMRI nude mice and subjected to fractionated irradiation with 5 × 2 Gy ± 250 µg/g metformin treatment (*n* = 10 animals for all groups). **A** Tumor volume was followed up, and means with upper and lower 95% confidence intervals are shown. *p*-values were calculated by two-way ANOVA with *post-hoc* Bonferroni-Holm correction. **B** Time to tumor volume > 200 mm^3^ was determined from growth curves shown in **A**, and group comparisons were performed by log-rank tests with *post-hoc* Bonferroni-Holm correction. **C** Schematic of tumor sampling for cytokine analyses shown in **D**, **E** (*n* = 6 animals for vehicle, 5 × 2 Gy, and metformin groups, *n* = 7 for 5×2 Gy + metformin group). **D** Cytokine levels as measured in lysates of tumor explants by multiplex ELISAs. Results from individual mice are shown with superimposed boxes (1st, 2nd, and 3rd quartiles). Asterisks indicate means, and whiskers depict extreme values. *p*-values were determined by Student’s *t*-tests wi*t*h Benjamini-Hochberg correction. **E** Subgroup analysis in the 5 × 2 Gy + metformin group separated into tumors with attenuated and without attenuated cytokine response. Tumor volumes and tumor weights were determined on the day of explantation, and group comparisons were performed by Student’s *t*-tests. **F** Gene set enrichment analysis (GSEA) of differentially expressed human transcripts in xenografts with attenuated cytokine response vs. tumors without attenuated cytokine response (MSigDB hallmark gene sets). Left panel: global enrichment plot, right panel: Hallmark gene sets with FDR *q*-values < 0.1.

In order to examine whether the addition of metformin can also radiosensitize resistant HNSCC in a clinically more relevant radiation setting, Cal 33 xenografts were subjected to conventionally fractionated radiotherapy (daily doses of 2 Gy). Cal 33 cells were used because SASP cytokine production in this cell line was already observed at radiation doses ≤ 4 Gy (other than in UPCISCC 040 cells which require doses > 4 Gy) for stimulation of cytokine production (Supplementary Fig. 4D–G). In this model, the combination of metformin plus radiotherapy again showed the best performance of all treatments (Fig. 6A, B). However, with regards to time-to-tumor-volume ≥ 200 mm^3^, the combination treatment was not significantly superior to radiotherapy alone (Fig. 6B). We, therefore, explanted the tumors at the end of treatment (Fig. 6C) and analyzed the impact of metformin on radiotherapy-induced SASP cytokine production by multiplex ELISAs. Unexpectedly, the tumors of the combined modality group showed a very heterogeneous response, and the addition of metformin did not abrogate radiotherapy-induced cytokine production in all samples. This was particularly evident for CXCL1, CXCL8, IL1α, and ILβ. (Fig. 6D). However, when these samples were dichotomized into tumors with and without attenuated cytokine response, the subgroup with the attenuated cytokine response displayed significantly decreased tumor volumes and tumor weights (Fig. 6E), indicating that the attenuation of SASP cytokine production by metformin administration was paralleled by improved treatment outcome. On the molecular level, GSEA of RNA sequencing data revealed that the tumors of the combined modality group with attenuated SASP cytokine production showed significant negative enrichment of gene sets involved in the regulation of proliferation, cell cycle control, reactive oxygen species detoxification, and DNA repair which concertedly may contribute to the improved radiotherapeutic outcome (Fig. 6F). Although the underlying reasons for the heterogeneous cytokine response in the combined modality group remain unclear, these results indicate that attenuation of radiation-induced cytokine expression by metformin and enhanced radiation-mediated tumor growth control in HNSCC xenotransplants are obviously associated.

### Clinical relevance of CXCR2 and its ligands in the TCGA HNSCC cohort and a retrospective in-house cohort of HNSCC patients

**Fig. 7:**
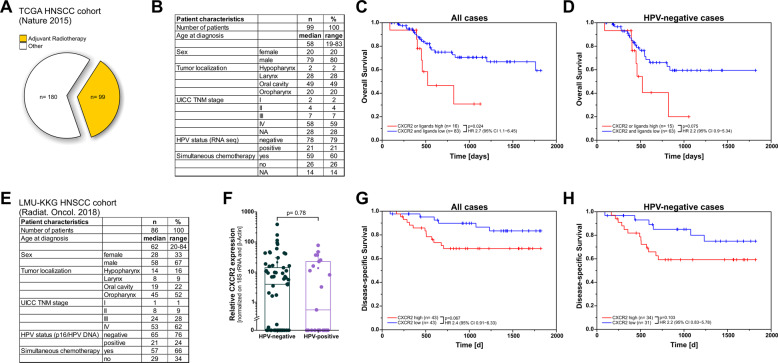
Clinical relevance of CXCR2 and its ligands in the TCGA HNSCC cohort and a retrospective in-house cohort of HNSCC patients (LMU-KKG HNSCC). A radio(chemo)therapy-treated subgroup of the TCGA HNSCC cohort (A–D) and a retrospective in-house cohort of HNSCC patients with post-operative radio(chemo)therapy (E–H) were used for Kaplan-Meier survival analyses dichotomized by mRNA expression levels of CXCR2 and/or its ligands. **A** Pie chart illustrating the subgroup of 99 out of 279 individuals of the TCGA HNSCC cohort receiving post-operative radio(chemo)therapy. **B** Patient characteristics, clinical data, and HPV status of the TCGA subgroup with post-operative radio(chemo)therapy. **C** Kaplan-Meier analysis of overall survival in the TCGA subgroup with post-operative radio(chemo)therapy (*n* = 99) stratified according to mRNA expression levels of CXCR2 and/or its ligands. Data were dichotomized using z-score ≥2 as cutoff. *p*-values were calculated by log-rank tests, 95% confidence intervals, and hazard ratios are shown. **D** Kaplan-Meier overall survival analysis as in **C** for HPV-negative cases only (*n* = 78). **E** Patient characteristics, clinical data, and HPV status of the LMU-KKG HNSCC cohort. **F** Boxplot showing relative CXCR2 mRNA expression levels in tumor samples as determined by qRT-PCR (relative standard curve quantification with normalization on a reference gene matrix of 18 S rRNA and β-Actin) for HPV-positive (*n* = 21) and HPV-negative (*n* = 65) cases of the LMU-KKG HNSCC cohort. Data of individual patients are shown, superimposed boxes represent 1st, 2nd, and 3rd quartiles, asterisks indicate means, and whiskers indicate SD. *p*-value was calculated by 2-sided exact Wilcoxon-Rank test. **G** Kaplan-Meier analysis of disease-specific survival in the KKG-LMU HNSCC cohort (*n* = 86). Data were dichotomized according to CXCR2 mRNA expression with median expression as cutoff. **H** Analogous analysis as in **G** for HPV-negative cases only (*n* = 65). *p*-values were calculated by log-rank tests, 95% confidence intervals, and hazard ratios are shown.

Having shown that the induction of an early senescence response paralleled by the upregulation of acute SASP cytokines and particularly ligands of the CXCR2 receptor upon radiotherapy is a therapeutically targetable determinant of radioresistance in preclinical models of HNSCC, we next examined the clinical relevance of CXCR2 and its ligands in HNSCC patients. To this end, we investigated a subgroup of the TCGA HNSCC cohort undergoing post-operative radio(chemo)therapy [11, 29] (Fig. 7A, B) and an in-house cohort of HNSCC patients receiving post-operative radio(chemo)therapy (Fig. 7E) [30]. Overexpression of CXCR2 and/or its ligands was associated with significantly impaired overall survival in the radio(chemo)therapeutic TCGA subcohort. The same trend was observed when HPV-negative cases only were analyzed, yet statistical significance was not reached (Fig. 7C, D). With shorter follow-up interval but more specific clinical endpoints, overexpression of CXCR2 was similarly associated with impaired disease-specific survival in our in-house HNSCC cohort (Fig. 7E–H).

## Discussion

In the present study, we systematically analyzed resistance against radiotherapy and its underlying mechanisms in a panel of human HNSCC cell lines and xenotransplants derived thereof. Integration of clonogenic survival data with molecular and functional data on DNA damage repair and different cell fate decisions identified early radiation-induced tumor cell senescence accompanied by NF-κB-dependent production of acute SASP cytokines as a pharmacologically targetable axis of radiotherapeutic treatment failure. According to our retrospective clinical analyses this should be of particular benefit for a relevant subset of HNSCC cases with increased expression of CXCR2 and/or its ligands and impaired clinical outcome.

Our results strengthen the role of cellular senescence as tumor promoting and therapy resistance driving force that has recently emerged [4, 23, 31]. Although senescent cells in irradiated cultures of radioresistant HNSCC cell lines disintegrated after a while, their NF-κB-dependent secretome potently supported clonogenic survival in a non-cell autonomous, paracrine manner. The repertoire of SASP cytokines produced by radioresistant HNSCC cells was different from the ones being produced by radiosensitive HNSCC cells or normal oral keratinocytes, respectively, and the ligands of the CXCR2 receptor showed the strongest correlation to radioresistance. Obviously, resistance-associated senescent tumor cell-derived cytokines complement the spectrum of senescent host cell-derived cytokines that have been described to orchestrate the pro-tumorigenic response in the context of a therapy-resistant niche [32, 33]. The mechanistic basis for the discrepant SASP profiles remains unclear so far. Nevertheless, it may be assumed that the genetic repertoire of the senescing cells as well as diverging functionality of fundamental processes involved in RNA transcription, (alternative) splicing, translation, and protein secretion are involved [27, 28, 34].

Findings that are in line with ours have been reported for cisplatin-treated melanoma models, and with a therapeutic perspective IL1α and CXCL8 were identified as crucial and pharmacologically targetable drivers [23]. In our radioresistant models of HNSCC, interference with NF-κB-dependent SASP production by administration of metformin showed promising therapeutic performance when combined with radiotherapy. Thus, “unSASPing” therapy-induced cancer cell senescence by so-called senomorphic agents appears to be an attractive multi-modality treatment approach with translational potential on several levels [34, 35]. With slightly different motivation but in principle along the same lines, elimination of senescing cancer cells by adding senolytic drugs to radio(chemo)therapy has been suggested as a “one-two-punch” treatment strategy [2, 36, 37]. Both, senomorphic as well as senolytic agent-augmented radiotherapy may be of particular interest for the treatment of HNSCC where curative concepts predominantly rely on adjuvant radiochemotherapy [14]. Of note, various SASP targeting and/or senolytic agents (i.e., neutralizing antibodies against IL1α, and/or CXCR2 ligands, NF-κB inhibitors, such as metformin, and senolytics, including ABT-263) have been in clinical use for years. Thus, toxicity profiles are well-known, and clinical testing should be implementable in a timely manner [7, 35]. Moreover, senotherapeutic agents would not only target therapy-induced senescent tumor cells but also therapy-induced senescent normal tissue cells. According to the current understanding, the induction of senescence in normal tissue is a major determinant of long-term side effects of radiotherapy, including hyposalivation/xerostomia or radiation-induced lung fibrosis, respectively [38, 39]. Hence, it may be speculated that these could also be alleviated by multi-modal senotherapeutic radiotherapy.

The xenogeneic nature of the model systems used in the present study brings several advantages, including the human origin and the possibility to distinguish tumor cell-derived from host cell-derived SASP production. Nevertheless, it bears the disadvantage of immunocompromised in vivo models. Given that SASP factors have been reported to shape immune cell recruitment and immune surveillance of tumors [40, 41], the question arises of how unSASPing therapy-induced senescence will affect the contribution of immune mechanisms to the overall treatment outcome. Interestingly, although a mouse model of SASP-deprived senescence-driven liver tumor formation showed reduced immune cell recruitment and immune cell-mediated tumor cell clearance, no enhanced tumor formation was observed [34]. These seminal findings need to be confirmed in truly therapeutic settings with fully immunocompetent model systems and long-term follow-up, in order to carefully examine the tumor immunological aspects of SASP-targeting, multi-modality anti-cancer therapy.

Finally, it is of pivotal importance to understand which subgroups of patients should be considered for such combined modality treatment strategies. Our retrospective clinical analyses suggest that HNSCC patients with elevated expression of CXCR2 and/or its ligands may particularly benefit — either from broad range SASP-targeting radio(chemo)therapy *via* interference with NF-κB function or more specifically from targeting distinct cytokine/receptor systems. Interestingly, the prognostic relevance and the therapeutic potential of CXCR2 and its ligands in HNSCC have very recently been described. However, this was basically attributed to metastasis formation, and immune evasion [42–44]. Our study links the CXCR2/ligand axis to resistance against radiotherapy. Its specific association with early radiation-induced tumor cell senescence and an NF-κB-dependent expression of acute SASP cytokines renders it an attractive target for mechanism-based combined modality radio(chemo)therapy whose translational potential deserves further investigation.

## Materials and methods

### Cell lines

HNSCC cell lines Cal 27, Cal 33, UDSCC 2, UPCISCC 040, UPCISCC 099, UPCISCC 131, UPCISCC 154 were purchased from ATCC (Manassas, VA, USA), DSMZ (Braunschweig, Germany), or obtained from Prof. Thomas Hoffmann (University of Ulm, Germany) and authenticated after retrieval by short tandem repeat (STR) typing (service by DSMZ) [45–47]. Cell lines were cultured in DMEM supplemented with 10% FCS, 100 U/ml penicillin, and 100 µg/ml streptomycin (all from Thermo Scientific, Schwerte, Germany) at 37 °C and 7.5% CO_2_. Human hTERT-immortalized keratinocytes OKF 6 were cultured in a 2 + 1 + 1 Keratinocyte-SFM/DMEM/F12 nutrient mixture, supplemented with 0.3 ng/ml rEGF, 35 µg/ml bovine pituitary extract, 155 µM CaCl_2_, 1.5 mM Glutamax, 25 U/ml penicillin, and 25 µg/ml streptomycin (Thermo Scientific) [48]. All cell lines were routinely tested negative for mycoplasma contamination. Cells were irradiated at the indicated doses using an RS225 X-ray cabinet (X-Strahl, Camberley, UK) operated at 200 kV/10 mA (Thoraeus filter, 1 Gy in 63 s).

### Primary human oral keratinocytes

Normal human oral keratinocytes were derived from 2 mm biopsies of non-attached gingiva of healthy volunteers as approved by the University Medical Center of Freiburg ethics committee. Cultures were established using a modified explant culture method and a defined keratinocyte medium (Promocell, Heidelberg, Germany), supplemented with 25 U/ml penicillin, and 25 µg/ml streptomycin. During the first 3–4 days, medium was supplemented with 5% human serum to enhance initial keratinocyte outgrowth. On days 10–12, cells were first passaged using Accutase (PAA, Coelbe, Germany). Irradiation was performed with adherent keratinocytes at passage 1 using ^137^Cs in a Gammacell 40 Exactor (Best Theratronics, Ottawa, Canada) at a dose rate of 0.65 Gy/min. Supernatants were harvested 96 h after irradiation and stored at −80 °C until cytokine analysis was performed.

### Conditioned medium

UPCISCC 040 cells were seeded at 2.5 × 10^3^–10^4^ cells/cm^2^ into T75 flasks and were irradiated at 8 Gy. After 6 days, culture supernatants were filtered (0.2 µm pore size) and frozen at −80 °C until further use. Conditioned medium of viable, non-irradiated cells served as control. For medium transfer experiments, 1 + 1 mixtures with fresh medium were employed.

### Drug treatment

Olaparib (Tocris/Biozol, Eching, Germany) was dissolved at 100 mM in DMSO (Sigma-Aldrich, Taufkirchen, Germany) and used at 100 nM in culture medium. zVAD-fmk (Bachem, Bubendorf, Switzerland) was dissolved at 20 mM in DMSO/ethanol (Sigma-Aldrich) and used at 50 µM final concentration in culture medium. Metformin (Sigma-Aldrich) was reconstituted to 500 mM in phosphate-buffered saline (PBS) and diluted to 5 mM in culture medium. Drugs were applied during medium change directly before irradiation and were not removed until the end of the experiment.

### siRNA transfection

Transfection of stealth^TM^ siRNA oligonucleotides (50 nM oligonucleotides, from Thermo Scientific, sequences are listed in Supplementary Table 1) was performed using Lipofectamine 2000 (Thermo Scientific) according to [49]. Knockdown efficiency was confirmed by qRT-PCR analyses 24 h after transfection, and cells were reseeded for clonogenic survival assays.

### Clonogenic survival

Clonogenicity was determined in colony formation assays as described previously [8]. Cells were seeded into six-well plates in a range of 1 × 10^1^–10^4^ cells/cm^2^, depending on cell line and irradiation dose. Before irradiation at 0–8 Gy, medium was changed to fresh medium or medium supplemented as indicated (conditioned medium or medium plus drugs). Colony formation was allowed for 14 days. Colonies with more than 50 cells were counted, and the percentage of surviving colonies was calculated by normalization to the plating efficiency of non-irradiated control cells. Survival curves were subjected to linear-quadratic fitting, and α/β-values were calculated.

### Flow cytometry

Flow cytometry was performed on an LSRII cytometer (BD Biosciences, Heidelberg, Germany), and data were analyzed with FACSDiva (BD Biosciences), or FlowJo v10 Software (Tree Star Inc., Ashland, OR, USA) respectively. For senescence detection, senescence-associated β-gal activity was measured upon lysosomal alkalinization by bafilomycin A1 treatment. Cells were seeded at 2.5 × 10^4^ cells/cm^2^ into 24-well plates, irradiated the next day at the indicated doses and incubated for 1–8 days. Cells were treated with 100 nM bafilomycin A1 (LC Laboratories, Woburn, MA, USA) for 1 h at 37 °C followed by staining with 5-dodecanoylaminofluorescein-di-β-galactopyranoside (C_12_FDG, Thermo Scientific) as described [50, 51]. Viable cells with high C_12_FDG and high SSC signal were considered senescent (Supplementary Fig. 1A). For quantification of apoptotic nuclei, cells were seeded at 2.5 × 10^4^ cells/cm^2^ into 24-well plates and irradiated upon adherence. After 1–4 days, trypsinized cells were fixed in 70% ethanol, stained with propidium iodide (PI) staining buffer (38 mM tri-sodium-citrate, 69 µM PI, 100 µg/ml RNAseA, all Sigma-Aldrich) and measured by flow cytometry as described [49]. For quantification of necrosis, PI exclusion staining was employed. Upon treatment, cells were harvested by trypsinization and resuspended in FACS staining buffer (BD Biosciences) supplemented with 5 µg/ml PI. After 5 min of incubation and washing in FACS staining buffer, cells with impaired plasma membrane integrity were identified by increased PI fluorescence.

### SDS-PAGE and westernblot analyses

Reducing gradient SDS-PAGE and Westernblot analyses of whole cell lysates (20–300 µg total protein per lane) were carried out as described [52]. Primary antibodies against lamin B1 (rabbit anti-human lamin B1 (Abcam # ab16048, Amsterdam, Netherlands)) and vinculin (mouse anti-human vinculin (Sigma-Aldrich #V9131, Taufkirchen, Germany)) were used, and detection and quantification of IRDye-conjugated secondary antibodies (LI-COR Biosciences, Bad Homburg, Germany) was performed with a LI-COR Odyssey scanner.

### Caspase activity tests

Effector caspase activity was measured in enzymatic assays with whole cell lysates as prepared for Westernblot experiments and the fluorogenic peptide Ac-DEVD-AMC (Bachem, Bubendorf, Switzerland) as described previously [52].

### Xenograft tumor models

All animal experiments were performed in accordance with ethical approval of the local Committee on the Ethics of Animal Experiments of the *Landesamt für Natur, Umwelt und Verbraucherschutz (LANUV), Regierungspraesidium Duesseldorf*. Mice were housed under standard conditions in individually ventilated cages with a 12 h day/night cycle and food and water *ad libitum* throughout the experiments. Mouse xenograft tumors were generated by a single subcutaneous injection of 50 µl growth factor reduced matrigel (Thermo Scientific) containing 0.5 × 10^6^ cells onto the hind limb of immunodeficient NMRI nude mice with subsequent incubation for 3–5 days before treatments were started. Animals carrying tumors smaller than 100 mm^3^ or bigger than 200 mm^3^ at the day of treatment start were excluded from experiments, and mice were randomized within cages into different treatment groups. Irradiation was applied under isoflurane (2–3%) anesthesia in daily fractions of 2 Gy in 5 mm tissue depth using a collimated beam (17 × 22 mm^2^) with an RS320 X-ray cabinet (X-Strahl, 300 kV, 10 mA, 0.5 mm Cu-filter, ∼1.53 Gy/min) [53]. Metformin was administered intraperitoneally at 250 µg/g in 100–150 µl PBS at the indicated time points. PBS injection served as vehicle control. Animals were inspected daily in a blinded manner by an independent investigator not involved in the treatment, and tumor size was evaluated three times per week. For tumor growth control experiments, animals were sacrificed when reaching a predefined health score (comprising critical tumor size ≥ 1500 mm^3^, critical weight loss, tumor ulceration, restriction of movement, and overall health performance). For qRT-PCR and multiplex cytokine analyses, animals were sacrificed on the last day of treatment; tumors were explanted, and immediately frozen at −80 °C. Total follow-up time ranged between 12 and 60 days.

### Immunohistochemical analysis of tumor cell senescence in xenograft tumors

Detection of senescent tumor cells was performed by immunohistochemical staining of senescence-associated β-gal in cryosections as described recently [39].

### Gene expression analyses by qRT-PCR

Cells were seeded at 2.5 × 10^4^ cells/cm^2^, treated as indicated and RNA was extracted using NucleoSpin RNA II Kit (Macherey & Nagel, Dueren, Germany) according to the manufacturer’s instructions. RNA of tumor explants was extracted using Trizol reagent (Thermo Scientific). RNA of formalin-fixed and paraffin-embedded tumor samples was extracted with the AllPrep DNA/RNA FFPE Kit (Qiagen, Hilden, Germany). 1 µg RNA was reversely transcribed using 200 U RevertAid reverse transcriptase in the presence of 5 μM random hexamers, 5 μM Oligo(dT)_18_, 500 μM dNTPs, and 1 U/μl Ribolock RNase inhibitor (all from Thermo Scientific) in a final volume of 20 µl. qRT-PCR analyses were performed on an LC480 qPCR-cycler (Roche Applied Science, Penzberg, Germany) as described [54]. Relative quantification was performed using the relative standard curve method or the ddC_T_-method. For normalization, a reference gene matrix was used. Primer sequences are listed in Supplementary Table 2.

### Gene expression analyses by RNA sequencing

Prior to library generation, RNA was quantified using the Qubit™ RNA BR Assay Kit with the Qubit Fluorometer (Thermo Scientific). RNA integrity was assessed by determining DV200 values (percentage of fragments > 200 nucleotides) using the 2100 Bioanalyzer (Agilent Technologies GmbH, Waldbronn, Germany) in combination with the Agilent RNA 6000 Nano Kit. RNA sequencing libraries were prepared with 100 ng input of total RNA using the QuantSeq 3′ mRNA-Seq Library Prep Kit FWD for Illumina (Lexogen, Vienna, Austria) following the manufacturer’s instructions for single indexing and good RNA quality. For library amplification, PCR cycles were determined with the PCR Add-on Kit for Illumina (Lexogen), and the individual libraries were amplified with 18 PCR cycles. The quality and quantity of the libraries were evaluated using the Quanti-iT PicoGreen dsDNA Assay Kit (Thermo Scientific) and the Bioanalyzer High Sensitivity DNA Analysis Kit (Agilent Technologies). Sequencing of libraries was performed on an Illumina HiSeq4000 platform with 150 bp paired-end mode while only forward reads were used for alignment to a multi-species reference (GrCH38and mm10) using STAR [55]. Aligned reads were counted at the gene level using htseq-count before importing into R using tximport. Only reads aligning human sequences with more than 100 reads across all samples were kept in the dataset. Variance-stabilized gene expression values were calculated with DESeq2 and used to generate a gene list ranked by log-fold changes for each comparison [56] which was subjected to gene set enrichment analysis (GSEA, linux command line version 4.0.3) in pre-ranked mode against MSigDB gene set collections (v7.0). RNA sequencing data shown in Fig. 6 have been deposited in NCBI’s Gene Expression Omnibus (GEO) with accession no. GSE185624. Computational code is available from the corresponding author upon request.

### Cytokine multiplex ELISA

Cytokine multiplex ELISA measurements with culture supernatants and tumor lysates were performed on a MAGPIX platform (Bio-Rad, Freiburg, Germany) using a Procartaplex Mix&Match panel (eBioscience/Thermo Scientific) consisting of the following analytes: CXCL1, CXCL5, CXCL8, CXCL9, CXCL10, CXCL11, CXCL12, CCL2, CCL3, CCL4, CCL5, CCL7, EGF, GM-CSF, IL1α, IL1β, IL6, MIF, PAI1, TNF, tPA, VEGFα. Cells were seeded at 1.75–2 ×10^4^ cells/cm^2^ in 6-well plates. Cells were treated as indicated, cell-free supernatants were collected after 1–4 d, and stored at −80 °C. For supernatants, dilutions of 1:5 in culture medium were used (total 50 µl). Tumor explants were lysed in 40 µl cell lysis buffer (Thermo Scientific) and 70 µg total protein were subjected to multiplex ELISAs according to the manufacturer’s protocol. A standard curve of seven dilutions (1:4) was used to derive concentrations from a five-parameter logistic equation [57]. Fitting was done with the ‘nls.lm’ function of the R package ‘minpack.lm’ with spurious measurements outside the standard curve approximated by log-linear interpolation of the closest two standard points.

### Survival analyses

Kaplan-Meier estimations were employed to analyze differences in survival, group comparisons were performed by log-rank tests, and hazard ratios (HR) with 95% confidence intervals (CI) were determined (R, package ‘survival’).

### HNSCC cohort of The Cancer Genome Atlas (TCGA)

Clinical patient characteristics and gene expression data of CXCR2 and its ligands CXCL1, CXCL2, CXCL3, CXCL5, CXCL6, CXCL7, CXCL8 were downloaded from cBioPortal [, 2015 #532; Grossman, 2016 #535; Cerami, 2012 #536 https://portal.gdc.cancer.gov]. The subgroup of patients who underwent post-operative radiotherapy was extracted (*n* = 99), patients who received radiotherapy for neoadjuvant, recurrence or palliative treatment were excluded, and follow-up was limited to 5 years [11]. For dichotomization, *z*-score ≥ 2 was used as a cutoff.

### Retrospective in-house cohort of HNSCC patients (LMU-KKG cohort)

Analyses were conducted according to the Declaration of Helsinki and were approved by the ethics committee of LMU (EA-448-13 and 17–116). Clinical data and tumor samples were collected, with patients’ written informed consent, in the framework of the clinical cooperation group “Personalized Radiotherapy in Head and Neck Cancer” [30, 58, 59]. Analyses were performed in the subgroup of patients who received post-operative radiotherapy at the Department of Radiation Oncology, LMU, Germany, between 2008 and 2013 (*n* = 86). HPV status of patients was determined by p16^INK4a^ immunohistochemistry combined with HPV DNA detection as described [60]. CXCR2 mRNA levels were determined by qRT-PCR, and the median expression level was used as cutoff for dichotomization. Disease-specific survival was calculated from the date of radiotherapy treatment start to the date of tumor-related death; in the absence of an event, patients were censored at the date of the last follow-up visit or the date of non–tumor-related death.

### Statistical analysis

Unless stated otherwise, data are presented as individual datapoints from independent experiments. For in vitro experiments, no statistical method was used to estimate sample size. The sample sizes were determined based on previous experiences and commonly accepted standards in the field. For in vivo experiments, group sizes were determined in consultation with a biostatistician according to the expected effect size, α-error of 0.05, and β-error of 0.2 (power 1-β = 0.8). Group comparisons of normally distributed data with similar variance were performed by two-way ANOVA and two-tailed, paired or unpaired Student’s *t*-tests as indicated. In the case of non-normal data distributions, group comparisons were calculated by two-sided exact Wilcoxon-Rank tests. Unsupervised hierarchical clustering and principal component analysis of z-normalized data were performed as described [8, 61]. Unsupervised hierarchical clustering of z-normalized data was performed using the Morpheus matrix visualization platform (https://software.broadinstitute.org/morpheus), principal component analyses and correlation analyses were carried out using OriginPro (OriginLab Ltd., Northhampton, MA, USA).

### Study approval

All animal experiments were performed in accordance with the Federation of European Laboratory Animal Science Associations (FELASA) guidelines and were approved by the local Committee on the Ethics of Animal Experiments of the *Landesamt für Natur, Umwelt und Verbraucherschutz (LANUV), Regierungspraesidium Duesseldorf*. Clinical investigations were conducted according to the Declaration of Helsinki and were approved by the local ethics committees of LMU and the University Medical Center of Freiburg. Patient data and tumor samples were collected in compliance with federal regulations and after obtaining written informed consent from the patients.

### Reporting summary

Further information on experimental design is available in the Nature Research Reporting Summary linked to this paper.

## Supplementary information

Supplemental Tables and Figures

Reporting summary

Supplemental movie

Coauthorship agreement

## Data Availability

Data are available from the corresponding author upon reasonable request. RNA sequencing data have been deposited in NCBI’s Gene Expression Omnibus (GEO) with accession no. GSE185624. Computational code is available from the corresponding author upon request.
