# Supplementary material for: Early senescence and production of senescence-associated cytokines are major determinants of radioresistance in head-and-neck squamous cell carcinoma
Source: Cell Death Dis. 2021 Dec 15;12(12):1162. doi: 10.1038/s41419-021-04454-5 (PMC8674332; doi:10.1038/s41419-021-04454-5)
Supplement: Supplementary file 1 — Supplemental Tables and Figures [file 41419_2021_4454_MOESM1_ESM.docx]

# **Early senescence and production of senescence-associated cytokines are major determinants of radioresistance in head-and-neck squamous cell carcinoma**

Ulrike Schoetz*, Diana Klein*, Julia Hess, Seyd Shnayien, Steffen Spoerl, Michael Orth, Samet Mutlu, Roman Hennel, Anja Sieber, Ute Ganswindt, Benedikt Luka, Andreas R. Thomsen, Kristian Unger, Verena Jendrossek, Horst Zitzelsberger, Nils Blüthgen, Claus Belka, Steffen Unkel, Bertram Klinger**, Kirsten Lauber**^§^

# **Supplemental Tables**

**Supplemental Table 1:** Key characteristics of the HNSCC cell line panel used in the present study. N.a. indicates information not available.

| Cell line | Origin | Age | Sex | TNM stage | Tumor grading | Previous treatment | HPV status | TP53 |
| --- | --- | --- | --- | --- | --- | --- | --- | --- |
| UDSCC2 | squamous cell carcinoma  hypopharynx, new primary | 58 | M | T1N2M0 | poorly differentiated | none | positive | WT |
| Cal27 | squamous cell carcinoma oral cavity new primary | 56 | M | N+ | poorly differentiated | none | negative | H193L |
| Cal33 | squamous cell carcinoma oral cavity new primary | 69 | M | n.a. | moderately differentiated | none | negative | R175H |
| UPCISCC040 | squamous cell carcinoma oral cavity new primary | 50 | M | T2N2 | moderately differentiated | none | negative | unclear |
| UPCISCC099 | squamous cell carcinoma oral cavity recurrence | 52 | M | T1N0 | moderately differentiated | none | negative | P177R |
| UPCISCC131 | squamous cell carcinoma oral cavity new primary | 73 | M | T2N2 | well differentiated | n.a. | negative | R248Q |
| UPCISCC154 | squamous cell carcinoma oral cavity new primary | 54 | M | T4N2 | poorly differentiated | n.a. | positive | WT |
| OKF6 | normal tissue  oral mucosal keratinocyte | 57 | M | healthy | healthy | none | negative | WT |

**Supplemental Table 2:** Oligonucleotides used for qRT-PCR analyses.

| **Target gene** | **Primer** | **Sequence** |
| --- | --- | --- |
| 18S rRNA | Forward | cggctaccacatccaaggaa |
|  | Reverse | AGCTGGAATTACCGCGGC |
| δ-ALAS | Forward | TCCACTGCAGCAGTACACTACCA |
|  | Reverse | ACGGAAGCTGTGTGCCATCT |
| β-Actin | Forward | CCTGGCACCCAGCACAATG |
|  | Reverse | GCCGATCCACACGGAGTACTT |
| β2-Microglobulin | Forward | TGCTCGCGCTACTCTCTCTTTC |
|  | Reverse | TCTCTGCTGGATGACGTGAGTAAAC |
| CXCL1 | Forward | AAGCTTGCCTCAATCCTGCAT |
|  | Reverse | TGGATTTGTCACTGTTCAGCATCT |
| CXCL2 | Forward | CGCATCGCCCATGGTTAA |
|  | Reverse | CAGTTGGATTTGCCATTTTTCAG |
| CXCL3 | Forward | CCCATGGTTCAGAAAATCATCG |
|  | Reverse | GTTGGTGCTCCCCTTGTTCA |
| CXCL5 | Forward | ATCCAGAAGCCCCTTTTCTAAAG |
|  | Reverse | TTTCCTTGTTTCCACCGTCC |
| CXCL6 | Forward | AAACTGCAGGTGTTCCCCG |
|  | Reverse | TTCTTGTTTCCACTGTCCAAAATTT |
| CXCL7 | Forward | CAAGTCGAAGTGATAGCCACACTG |
|  | Reverse | TCTGGGAGCATCTGGGTCC |
| CXCL8 | Forward | TGGCAGCCTTCCTGATTTCT |
|  | Reverse | TGCACTGACATCTAAGTTCTTTAGCA |
| CXCL10 | Forward | TGGCATTCAAGGAGTACCTCTCT |
|  | Reverse | GTAGCAATGATCTCAACACGTGG |
| CXCL11 | Forward | TGTTCAAGGCTTCCCCATGT |
|  | Reverse | GAGGCTTTCTCAATATCTGCCACT |
| CXCL12 | Forward | TGCTGGTCCTCGTGCTGAC |
|  | Reverse | TGGCAACATGGCTTTCGAA |
| CXCR2 | Forward | AAGCCCAGCGACCCAGTC |
|  | Reverse | AGGGATTCTGGTTCACATGGG |
| CCL1 | Forward | GCTTGCTGCTAGCTGGGATGT |
|  | Reverse | CTCCGCAAATGAGAAGCAACA |
| CCL3 | Forward | CACCATGGCTCTCTGCAACC |
|  | Reverse | AATCTGCCGGGAGGTGTAGC |
| CCL8 | Forward | GCTGGAGAGCTACACAAGAATCAC |
|  | Reverse | GCCCCGTTTGGTCTTGAA |
| CCL13 | Forward | AGGCTGAAGAGCTATGTGATCACC |
|  | Reverse | CCTTGCCCAGTTTGGTTCTG |
| CCL25 | Forward | GAGCGGGAGCTGCAATCTG |
|  | Reverse | GGGTTCCCACACACCTTCCT |
| IL1α | Forward | GCTTCCTGAGCAATGTGAAATACA |
|  | Reverse | CAAATTTCACTGCTTCATCCAGATT |
| IL1β | Forward | CCTGAGCTCGCCAGTGAAAT |
|  | Reverse | TTTAGGGCCATCAGCTTCAAAG |
| IL6 | Forward | GGTACATCCTCGACGGCATCT |
|  | Reverse | AGTGCCTCTTTGCTGCTTTCAC |
| EGF | Forward | GGGCCCACTACTACAGGACTCAG |
|  | Reverse | GGCAAGAGGACATCCCATCC |
| GM-CSF | Forward | GTAGAGACACTGCTGCTGAGATGAA |
|  | Reverse | CTCCTGGAGGTCAAACATTTCTG |
| HGF | Forward | ACTTCCATTCACTTGCAAGGCT |
|  | Reverse | CTCCACTTGACATGCTATTGAAGG |
| MIF | Forward | TGCACAGCATCGGCAAGAT |
|  | Reverse | ATGTAGACCCTGTCCGGGCT |
| VEGFa | Forward | GGCAGAATCATCACGAAGTGGT |
|  | Reverse | GGGTCTCGATTGGATGGCA |
| ATM | Forward | CTGGAAGAAGCACAAGTATTCTGGG |
|  | Reverse | TGGGATTGTTCGCTGCACA |
| ATR | Forward | GGTCACCACCAGACAGCCTAC |
|  | Reverse | GAACATCACCCTTGGACCAGA |
| BLM | Forward | ATGGACATCGCCAGCTGAAG |
|  | Reverse | GCAAAGTAGTGGGAAGATACGGG |
| BRCA1 | Forward | GCTATTTCTGGGTGACCCAGTC |
|  | Reverse | GATTCTCTTGCTCGCTTTGGA |
| BRCA2 | Forward | GTTTGTGAAGGGTCGTCAGACA |
|  | Reverse | AACTAAGGGTGGGTGGTGTAGCT |
| BRIP1 | Forward | GTGTGCTGTAGCCTCATTAATTGG |
|  | Reverse | GTTAAGAGCATTTCATTTCCACTCC |
| CHEK1 | Forward | GTTAAGAGCATTTCATTTCCACTCC |
|  | Reverse | GAGGTTATCCCTTTCATCCAACAG |
| CHEK2 | Forward | CTCTTGGAAGTGGTGCCTGTG |
|  | Reverse | GGGTCTGCCTCTCTTGCTGA |
| DCLRE1C | Forward | CGGAGCCAAAGTATAAACCACTG |
|  | Reverse | TCATCTTCCTCCTCTGAGTCT |
| DNA2 | Forward | TTCCTTAATACAGACAAGGTTCCAGC |
|  | Reverse | CATCCAGCCTTAACAAAAATGGAG |
| EXO1 | Forward | AGCTACGCTGGGCAATATGTTG |
|  | Reverse | AATGGGCAGGCATAGCAGTG |
| FEN1 | Forward | GTGTCCCAAAGGCCAGTCATC |
|  | Reverse | GGGCCACATCAGCAATTAGTTT |
| KU70 | Forward | GGGCTCCTTGGTGGATGAGT |
|  | Reverse | GCTTCCAGAACCTTCATTATCGTG |
| KU80 | Forward | GAGCATAGACTGCATCCGAGC |
|  | Reverse | CTGAAAACTTAATGGCTTCTTCCC |
| LIG4 | Forward | AAGCCTTGGAGCTTCGGTTT |
|  | Reverse | TCTGCAACACGACTATGATCTTCC |
| MRE11A | Forward | TGGAGAAGATTGAAGAAATGCTTGA |
|  | Reverse | CCACTATAGTCCACTCGCAGTCG |
| NBN | Forward | CAGGAAAACTTCCACACATCATTG |
|  | Reverse | TTTGTACCTCCATTTCCTGCCTTA |
| NHEJ1 | Forward | AGCTAGTCCTTCCCTGGTCTCC |
|  | Reverse | CCCATCAGAGGACGAATCAAAT |
| PALB2 | Forward | TGTATAATCTCGTGTGTGTAGCTTTGG |
|  | Reverse | CAATGCCCTGATCTCTCTGATTTC |
| PARP1 | Forward | GCCCGTGACAGGCTACATGT |
|  | Reverse | TTGGGTCTCCCTGAGACGTATG |
| PARP2 | Forward | TGGGTGGGAATCTTGAGCC |
|  | Reverse | CTTTCCCAAACATGTAACCTGTGA |
| PRKDC | Forward | AACAACTTTATGGTGGCCATGG |
|  | Reverse | ACTGGCAGAAACTGTGTAGCGG |
| RAD50 | Forward | CGAAGTACCTATCGTGGACAAGATATT |
|  | Reverse | TCGGCATCAGACCGTATTTCTAT |
| RAD51 | Forward | GCAGTGGCTGAGAGGTATGGTC |
|  | Reverse | TGGTGGTCTGTGTTGAACGC |
| RAD52 | Forward | CAGCAGAAGAGTACCAGGCCAT |
|  | Reverse | TCAATGTAGCACACCTTCTGGC |
| RAD54 | Forward | GAGTATGAAGCAGAGAAGAAGGGACT |
|  | Reverse | CCAGTTGGTATGTTGAAACGCAT |
| RBBP8 | Forward | CTGTAATAGATACAAAGGATGGCAGTCA |
|  | Reverse | AACCAATGTACAGTCCATGTCCACT |
| RPA1 | Forward | AGGCGACAAGCCGGACTAC |
|  | Reverse | AGGCTTGGTACATGCAGTTCTCTT |
| XRCC1 | Forward | GAGCTGAGGAGGGTGGCAG |
|  | Reverse | CGTGTTCTCATCCGTGGAGC |
| XRCC2 | Forward | AAGATTCACCTGTGCATGGTGAT |
|  | Reverse | ACATCGTGCTGTTAGGTGATAAAGC |
| XRCC3 | Forward | CCGAGGTCTGGCACTTGCT |
|  | Reverse | GCTGCAGTGCTGTAAGGATGC |
| XRCC4 | Forward | GGACATCAAACAAGAAGGGGAA |
|  | Reverse | GGATCTCGGTCAGCAGTCATTT |
|  |  |  |
| PARP2 | siRNA | AUGUCAGCAAAGUAGAUUCCUUUCC |
| Scramble | siRNA | Stealth RNAi™ siRNA Negative Control, Med GC, Thermo Scientific |

# **Supplemental Figures**

**
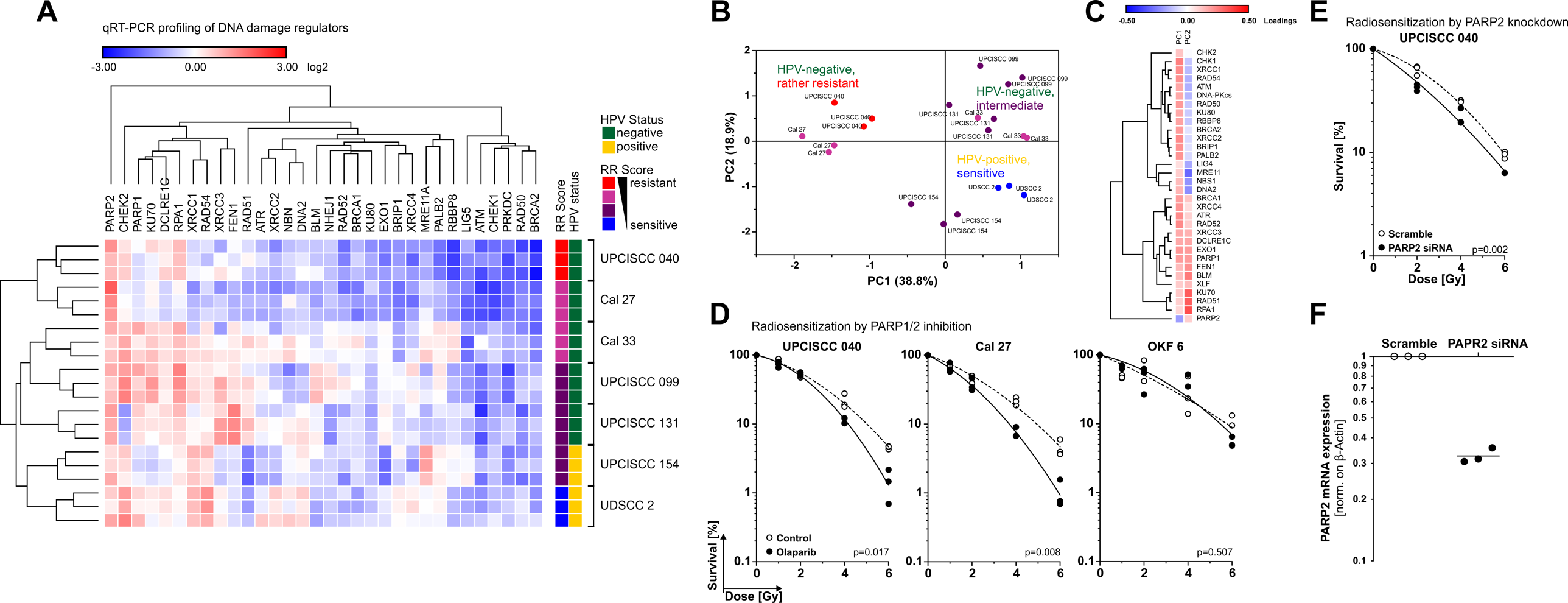
**

**Supplemental Figure 1:** **mRNA expression analysis of DNA double strand break repair regulators identifies PARP2 as a potential candidate of radiosensitization in distinct HNSCC cell lines.** **(A)** Basal gene expression levels of DNA damage response factors as measured by qRT-PCR (standard curve method; normalization on a reference gene matrix consisting of 18S rRNA, β2-Microglobulin, and δ-ALAS; calibration on expression levels of OKF6 cells; three biological replicates per cell line). Expression values (log 2-transformed) and samples were subjected to unsupervised hierarchical clustering resulting in two major clusters (UPCISCC040 and Cal27 vs. all others). **(B)** PCA of the data shown in (A). **(C)** Unsupervised hierarchical clustering of the loadings of the input variables on PC1 and PC2. **(D)** Targeted radiosensitization of PARP2 high expressing cell lines by pharmacological PARP1/2 inhibition as determined by clonogenic survival assays. Cells were treated with 100 nM olaparib directly before irradiation at doses between 0 and 6 Gy. Data points of three individual replicates are shown with linear-quadratic fitting functions superimposed. *p*-values were calculated by two-way ANOVA. hTERT-immortalized OKF6 keratinocytes served as normal tissue control cells. **(E)** Targeted radiosensitization of PARP2 high expressing UPCISCC040 cells by siRNA-mediated knockdown of PARP2 expression. Cells were transfected with 50 nM PARP2-specific siRNA oligonucleotide or a scramble control 24 h before irradiation at doses between 0 and 6 Gy. Three individual replicates are shown with linear-quadratic fitting functions superimposed. *p*-values were calculated by two-way ANOVA. **(F)** Knockdown efficiency in experiments shown in (E) was confirmed by qRT-PCR analysis of PARP2 expression (normalized on β-actin, calibrated on scramble controls). Three biological replicates and superimposed means are shown.


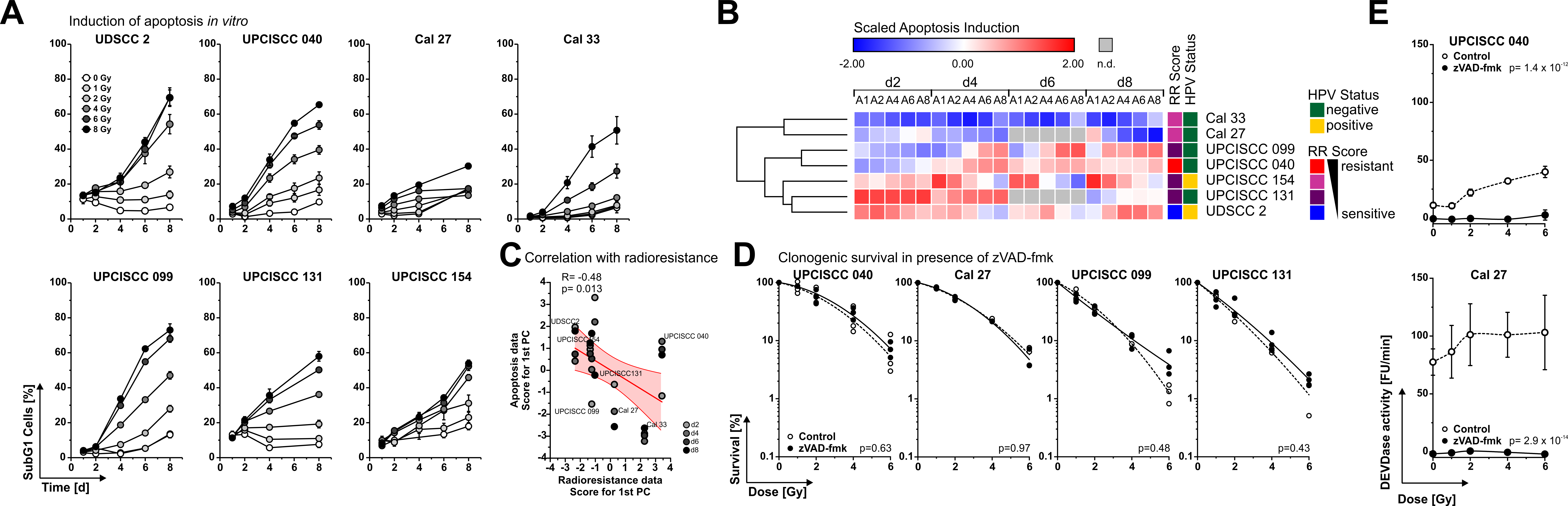


**Supplemental Figure 2: Induction of apoptosis negatively correlates with radioresistance, but inhibition of apoptosis by the poly-caspase inhibitor zVAD-fmk has no significant effect on clonogenic survival upon irradiation *in vitro*. (A)** Time-course of apoptosis induction after irradiation at 0-8 Gy as measured by flow cytometric detection of sub G1 cells. Means and SD of triplicates are depicted. **(B)** Unsupervised hierarchical clustering of z-normalized apoptosis data from (A, n.d. indicates not done). **(C)** Correlation analysis of the scores of the 1st PC of radioresistance (Fig. 1C) with the scores of the 1st PC of apoptosis induction at different time points (day 2 - day 8) with superimposed linear regression ± upper and lower 95% confidence intervals. Pearson's R and *p*-value are indicated. **(D)** Inhibition of apoptosis by zVAD-fmk in clonogenic survival assays. Four cell lines of varying radioresistance were treated with 50 µM zVAD-fmk directly before irradiation at 0-6 Gy. Results of three individual experiments are shown, *p*-values were calculated by two-way ANOVA. **(E)** Effectivity of zVAD-fmk treatment was confirmed by caspase [DEVDase] activity measurements on d8 after irradiation (20 µg total protein per sample, means ± SD of triplicates are shown), *p*-values were calculated by two-way ANOVA.

**
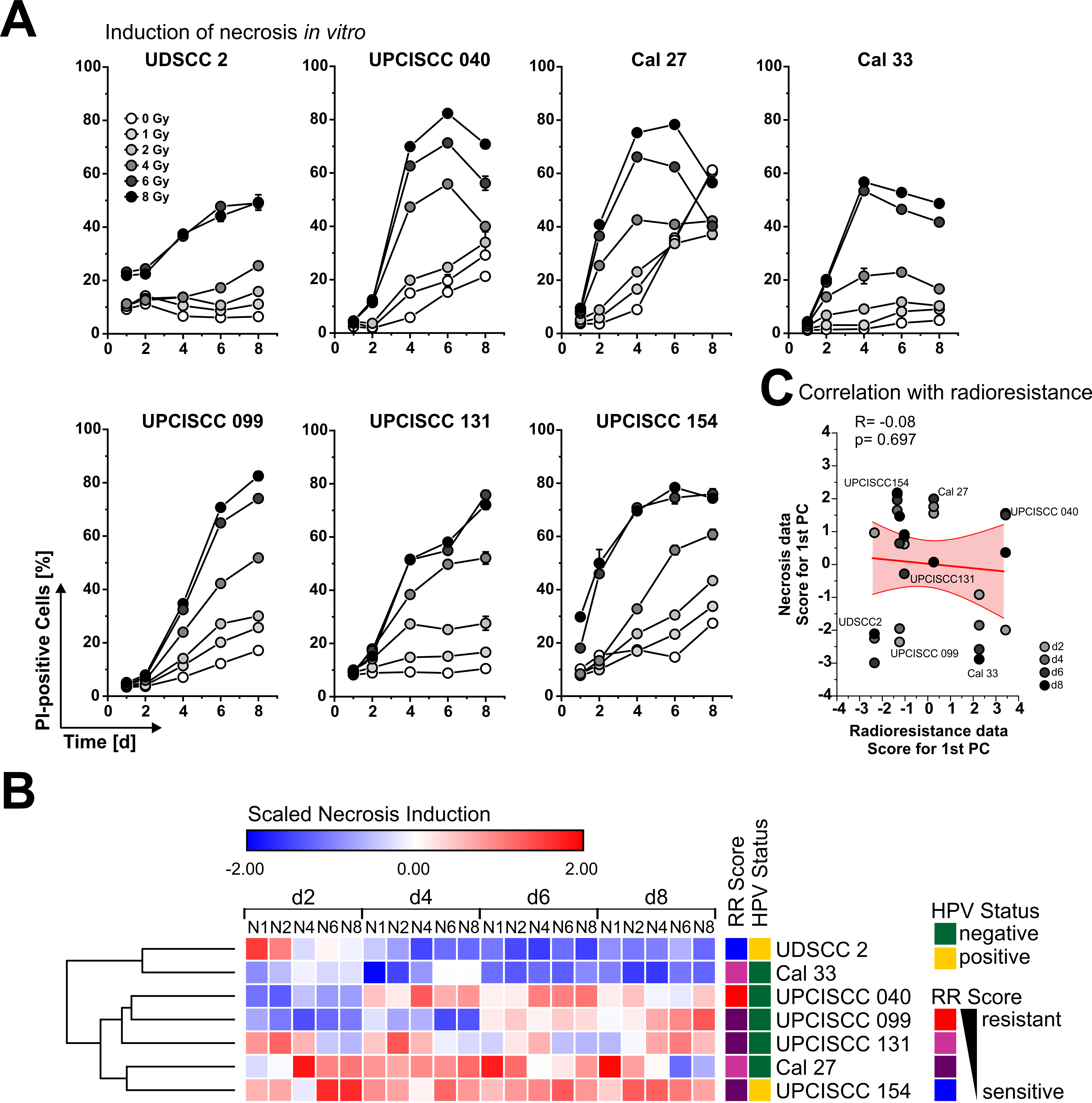
**

**Supplemental Figure 3: Induction of necrosis does not significantly correlate with radioresistance. (A)** Time-course of necrosis induction after irradiation at 0-8 Gy as measured by flow cytometric detection of propidium iodide-positive cells. Means and SD of triplicates are depicted. **(B)** Unsupervised hierarchical clustering of z-normalized necrosis data from (A). **(C)** Correlation analysis of the scores of the 1st PC of radioresistance (Fig. 1C) with the scores of the 1st PC of necrosis induction at different time points (day 2 ‑ day 8) with superimposed linear regression ± upper and lower 95% confidence intervals. Pearson's R and *p*-value are indicated.

**
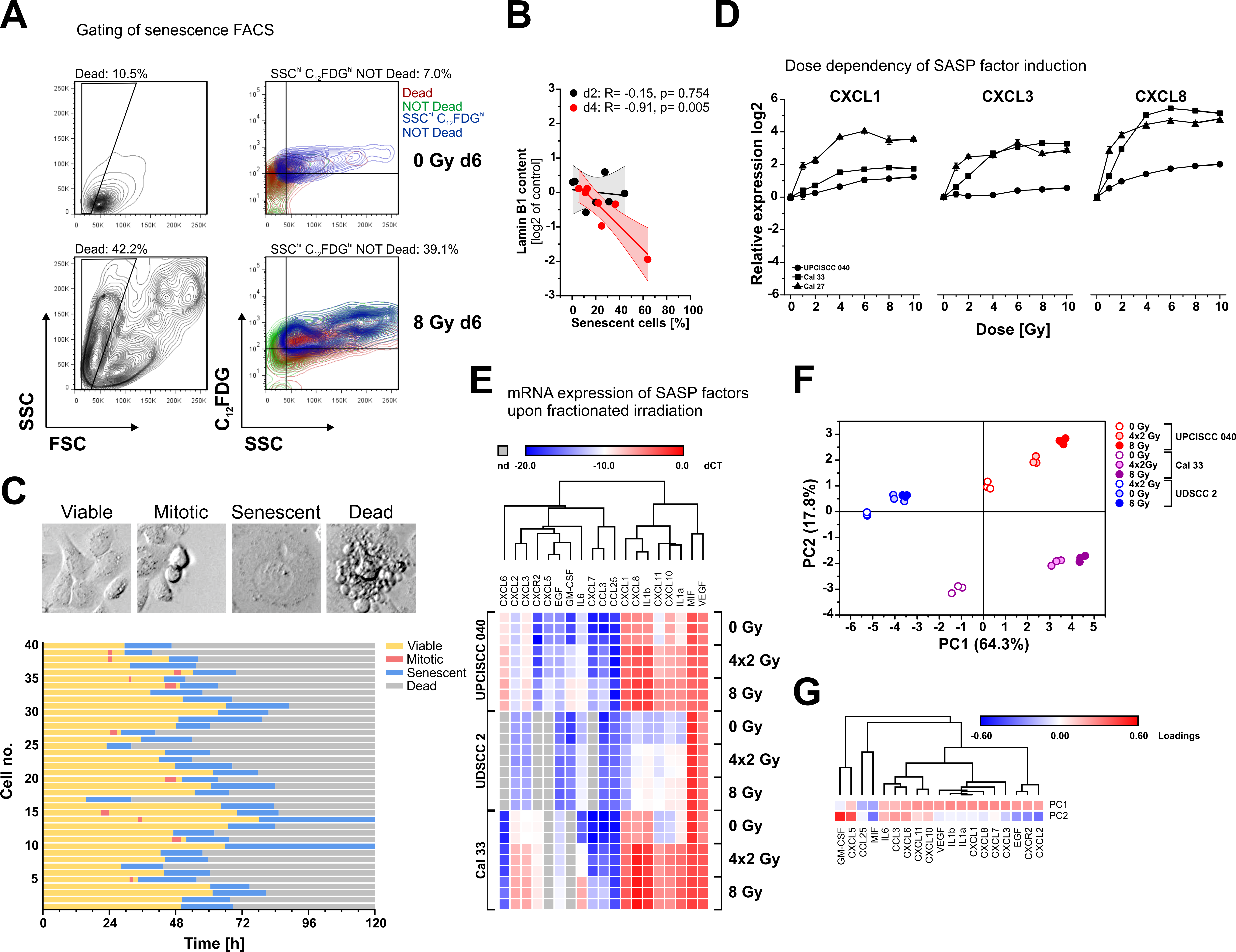
**

**Supplemental Figure 4: Induction of senescence and SASP production *in vitro* upon irradiation with different doses and fractionation regimens.** **(A)** Gating strategy for the detection of senescent cells by flow cytometric staining of senescence-associated β-gal. Cells with increased granularity (SSC^high^) and increased β-gal activity (C_12_-FDG^high^) outside the FSC-SSC "dead cells" gate were considered senescent. **(B)** Correlation of lamin B1 content as determined by Westernblot analysis and the percentage of senescent cells as determined by flow cytometric staining of senescence-associated β-gal (Fig. 2). Results obtained on day 2 and day 4 upon irradiation at 8 Gy are shown. Lamin B1 content was normalized to vinculin and calibrated on the respective 0 Gy-treated controls. Every dot represents one HNSCC cell line. **(C)** Cell fate tracking in time-lapse microscopy data (**Suppl. movie**). Forty UPCISCC040 cells with intermittent senescent morphology were randomly picked and tracked from day 0 - day 4 upon irradiation at 4 Gy. Classification as viable, mitotic, senescent, or dead was performed according to the reference morphologies shown in the upper image panel. **(D)** qRT-PCR analysis of CXCL1, CXCL3, and CXCL8 expression in UPCISCC040, Cal27, and Cal33 cells upon irradiation at doses from 0-10 Gy. **(E)** mRNA expression of CXCR2 and SASP cytokines as measured by qRT-PCR after single dose (1x 8 Gy) or fractionated (4x 2 Gy) irradiation (Standard curve method; normalization on a reference gene matrix of 18S rRNA, β2-Microglobulin, and δ-ALAS; three biological replicates per cell lines). Unsupervised hierarchical clustering of dCT values, grey squares indicate no detection (nd). **(F)** PCA of the data shown in (C). **(G)** Unsupervised hierarchical clustering of the loadings of the input variables on PC1 and PC2.
