# Supplementary material for: Early senescence and production of senescence-associated cytokines are major determinants of radioresistance in head-and-neck squamous cell carcinoma
Source: Cell Death Dis. 2021 Dec 15;12(12):1162. doi: 10.1038/s41419-021-04454-5 (PMC8674332; doi:10.1038/s41419-021-04454-5)
Supplement: Supplementary file 3 — Supplemental movie [file 41419_2021_4454_MOESM3_ESM.pptx]

## Slide 1
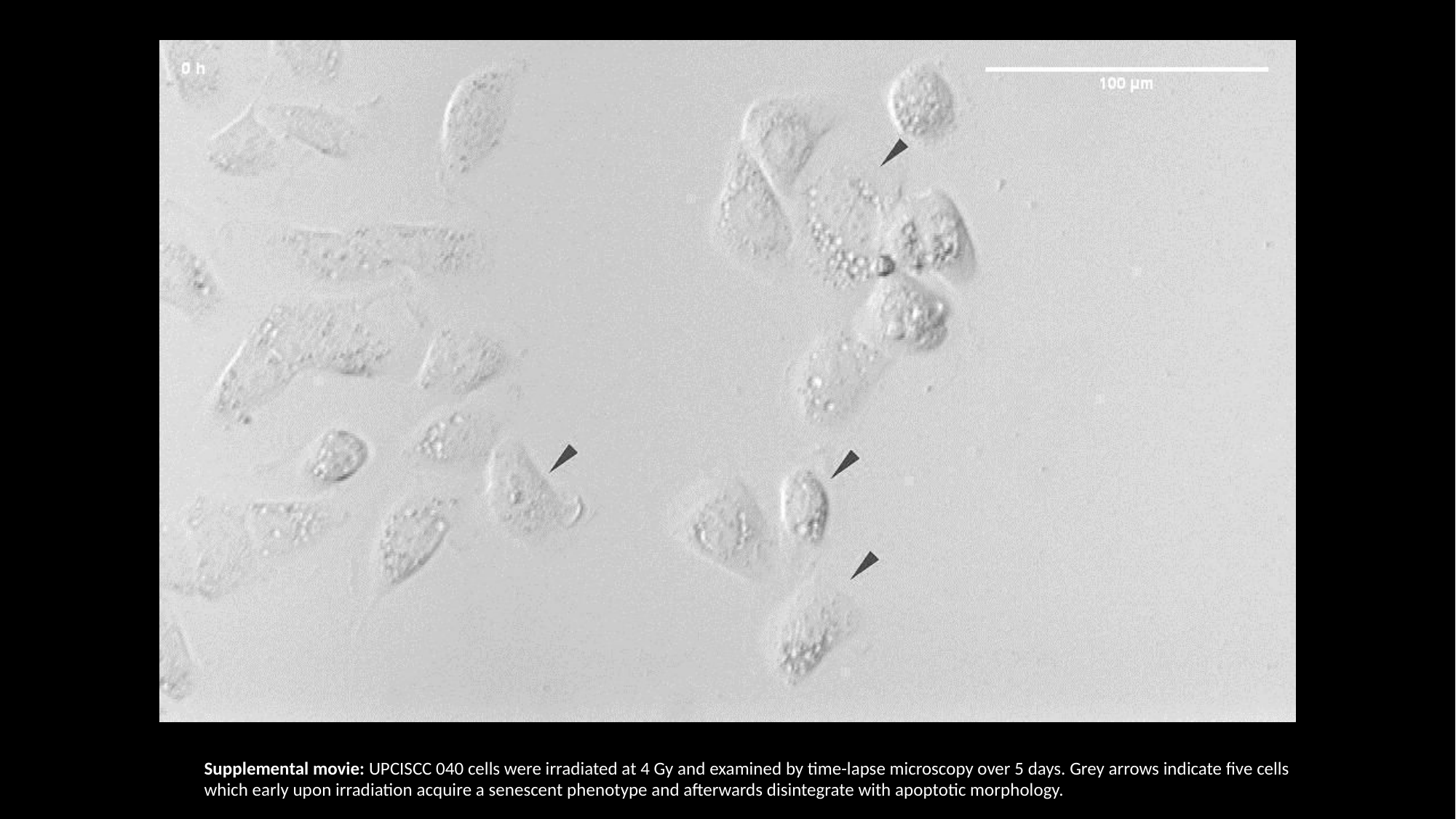

Supplemental movie: UPCISCC 040 cells were irradiated at 4 Gy and examined by time-lapse microscopy over 5 days. Grey arrows indicate five cells which early upon irradiation acquire a senescent phenotype and afterwards disintegrate with apoptotic morphology.
