# Supplementary material for: Early senescence and production of senescence-associated cytokines are major determinants of radioresistance in head-and-neck squamous cell carcinoma
Source: Cell Death Dis. 2021 Dec 15;12(12):1162. doi: 10.1038/s41419-021-04454-5 (PMC8674332; doi:10.1038/s41419-021-04454-5)
Supplement: Supplementary file 4 — Coauthorship agreement [file 41419_2021_4454_MOESM4_ESM.pdf]

Von: Lauber, Kirsten <Kirsten.Lauber@med.uni-muenchen.de>
Gesendet: Donnerstag, 25. November 2021 12:25
An: Lauber, Kirsten <Kirsten.Lauber@med.uni-muenchen.de>
Cc: ulrike.schoetz@staff.uni-marburg.de; seyd.shnayien@gmail.com; diana.klein@uk-essen.de; steffen.spoerl@ukr.de; bertram.klinger@charite.de; julia.hess@helmholtz-muenchen.de; Orth, Michael Dr.rer.nat. <Michael.Orth@med.uni-muenchen.de>; Galabov, Radostin Dr.rer.nat. <Radostin.Galabov@med.uni-muenchen.de>; Hennel, Roman Dr.rer.nat. <Roman.Hennel@med.uni-muenchen.de>; benedikt.luka@uniklinik-freiburg.de; anja.sieber@charite.de; ute.ganswindt@i-med.ac.at; andreas.thomsen@uniklinik-freiburg.de; verena.jendrossek@uk-essen.de; Belka, Claus Prof.Dr. <Claus.Belka@med.uni-muenchen.de>; nils.bluethgen@charite.de; unger@helmholtz-muenchen.de; steffen.unkel@med.uni-goettingen.de; zitzelsberger@helmholtz-muenchen.de
Betreff: CDDIS-21-2273R: Agreement to include Samet Mutlu into list of coauthors
Priorität: Hoch

Dear coauthors,

regarding our provisionally accepted CDDIS-21-2273 manuscript: Please confirm your agreement that Samet Mutlu is included into the list of coauthors. This is required by the journal standards. Samet has contributed the additional data on necrosis induction during revision (novel Fig. S3).

Please reply to this email simply with: Yes, agree.

Thanks

Kirsten

-----Ursprüngliche Nachricht-----
Von: Ulrike Schötz <ulrike.schoetz@staff.uni-marburg.de>
Gesendet: Donnerstag, 25. November 2021 12:28
An: Lauber, Kirsten <Kirsten.Lauber@med.uni-muenchen.de>
Cc: seyd.shnayien@gmail.com; diana.klein@uk-essen.de; steffen.spoerl@ukr.de; bertram.klinger@charite.de; julia.hess@helmholtz-muenchen.de; Orth, Michael Dr.rer.nat. <Michael.Orth@med.uni-muenchen.de>; Galabov, Radostin Dr.rer.nat. <Radostin.Galabov@med.uni-muenchen.de>; Hennel, Roman Dr.rer.nat. <Roman.Hennel@med.uni-muenchen.de>; benedikt.luka@uniklinik-freiburg.de; anja.sieber@charite.de; ute.ganswindt@i-med.ac.at; andreas.thomsen@uniklinik-freiburg.de; verena.jendrossek@uk-essen.de; Belka, Claus Prof.Dr. <Claus.Belka@med.uni-muenchen.de>; nils.bluethgen@charite.de; unger@helmholtz-muenchen.de; steffen.unkel@med.uni-goettingen.de; zitzelsberger@helmholtz-muenchen.de
Betreff: Re: CDDIS-21-2273R: Agreement to include Samet Mutlu into list of coauthors

Yes, agree.

Ulrike Schötz

Von: Klein, Diana <Diana.Klein@uk-essen.de>
Gesendet: Donnerstag, 25. November 2021 12:40
An: Lauber, Kirsten <Kirsten.Lauber@med.uni-muenchen.de>
Cc: ulrike.schoetz@staff.uni-marburg.de; seyd.shnayien@gmail.com; steffen.spoerl@ukr.de; bertram.klinger@charite.de; julia.hess@helmholtz-muenchen.de; Orth, Michael Dr.rer.nat. <Michael.Orth@med.uni-muenchen.de>; Galabov, Radostin Dr.rer.nat. <Radostin.Galabov@med.uni-muenchen.de>; Hennel, Roman Dr.rer.nat. <Roman.Hennel@med.uni-muenchen.de>; benedikt.luka@uniklinik-freiburg.de; anja.sieber@charite.de; ute.ganswindt@i-med.ac.at; andreas.thomsen@uniklinik-freiburg.de; Jendrossek, Verena <Verena.Jendrossek@uk-essen.de>; Belka, Claus Prof.Dr. <Claus.Belka@med.uni-muenchen.de>; nils.bluethgen@charite.de; unger@helmholtz-muenchen.de; steffen.unkel@med.uni-goettingen.de; zitzelsberger@helmholtz-muenchen.de
Betreff: AW: CDDIS-21-2273R: Agreement to include Samet Mutlu into list of coauthors

Yes, agree.

LG, Diana

Von: Klinger, Bertram <bertram.klinger@charite.de>
Gesendet: Donnerstag, 25. November 2021 13:56
An: Lauber, Kirsten <Kirsten.Lauber@med.uni-muenchen.de>
Betreff: Re: [ext] CDDIS-21-2273R: Agreement to include Samet Mutlu into list of coauthors [signed OK]
Priorität: Hoch

Yes, agree

Best wishes

Bertram Klinger

Von: Unkel, Steffen <steffen.unkel@med.uni-goettingen.de>
Gesendet: Donnerstag, 25. November 2021 12:44
An: Lauber, Kirsten <Kirsten.Lauber@med.uni-muenchen.de>
Betreff: AW: CDDIS-21-2273R: Agreement to include Samet Mutlu into list of coauthors

Yes, I do agree.

Steffen Unkel

Von: Seyd Shnayien <seyd.shnayien@gmail.com>
Gesendet: Donnerstag, 25. November 2021 16:14
An: Galabov, Radostin Dr.rer.nat. <Radostin.Galabov@med.uni-muenchen.de>
Cc: Lauber, Kirsten <Kirsten.Lauber@med.uni-muenchen.de>; ulrike.schoetz@staff.uni-marburg.de; diana.klein@uk-essen.de; steffen.spoerl@ukr.de; bertram.klinger@charite.de; julia.hess@helmholtz-muenchen.de; Orth, Michael Dr.rer.nat. <Michael.Orth@med.uni-muenchen.de>; Hennel, Roman Dr.rer.nat. <Roman.Hennel@med.uni-muenchen.de>; benedikt.luka@uniklinik-freiburg.de; anja.sieber@charite.de; ute.ganswindt@i-med.ac.at; andreas.thomsen@uniklinik-freiburg.de; verena.jendrossek@uk-essen.de; Belka, Claus Prof.Dr. <Claus.Belka@med.uni-muenchen.de>; nils.bluethgen@charite.de; unger@helmholtz-muenchen.de; steffen.unkel@med.uni-goettingen.de; zitzelsberger@helmholtz-muenchen.de
Betreff: Re: CDDIS-21-2273R: Agreement to include Samet Mutlu into list of coauthors

Yes, agree.

Von: Dr. Andreas Thomsen <andreas.thomsen@uniklinik-freiburg.de>
Gesendet: Donnerstag, 25. November 2021 15:48
An: Lauber, Kirsten <Kirsten.Lauber@med.uni-muenchen.de>
Betreff: AW: CDDIS-21-2273R: Agreement to include Samet Mutlu into list of coauthors

Yes, agree

Von: Steffen Spoerl <Steffen.Spoerl@klinik.uni-regensburg.de>
Gesendet: Donnerstag, 25. November 2021 15:23
An: Lauber, Kirsten <Kirsten.Lauber@med.uni-muenchen.de>
Betreff: Aw: [EXT] CDDIS-21-2273R: Agreement to include Samet Mutlu into list of coauthors

Dear Kisten,
of course I agree to include Samet Mutlu in the list of co-authors!
Best
Steffen

Von: Galabov, Radostin Dr.rer.nat. <Radostin.Galabov@med.uni-muenchen.de>
Gesendet: Donnerstag, 25. November 2021 13:51
An: Lauber, Kirsten <Kirsten.Lauber@med.uni-muenchen.de>
Cc: ulrike.schoetz@staff.uni-marburg.de; seyd.shnayien@gmail.com; diana.klein@uk-essen.de; steffen.spoerl@ukr.de; bertram.klinger@charite.de; julia.hess@helmholtz-muenchen.de; Orth, Michael Dr.rer.nat. <Michael.Orth@med.uni-muenchen.de>; Hennel, Roman Dr.rer.nat. <Roman.Hennel@med.uni-muenchen.de>; benedikt.luka@uniklinik-freiburg.de; anja.sieber@charite.de; ute.ganswindt@i-med.ac.at; andreas.thomsen@uniklinik-freiburg.de; verena.jendrossek@uk-essen.de; Belka, Claus Prof.Dr. <Claus.Belka@med.uni-muenchen.de>; nils.bluethgen@charite.de; unger@helmholtz-muenchen.de; steffen.unkel@med.uni-goettingen.de; zitzelsberger@helmholtz-muenchen.de
Betreff: AW: CDDIS-21-2273R: Agreement to include Samet Mutlu into list of coauthors

Yes, agree

Von: Dr. Benedikt Luka <benedikt.luka@uniklinik-freiburg.de>
Gesendet: Donnerstag, 25. November 2021 13:08
An: Lauber, Kirsten <Kirsten.Lauber@med.uni-muenchen.de>
Betreff: AW: CDDIS-21-2273R: Agreement to include Samet Mutlu into list of coauthors

Yes, agree.

UNIVERSITÄTSKLINIKUM FREIBURG
Department f. Zahn-, Mund- u. Kieferheilkunde
Klinik für Zahnerhaltungskunde und Parodontologie

Dr. Benedikt Luka
Wissenschaftlicher Mitarbeiter/Zahnarzt
der Stiftungsprofessur für Kariesforschung

Hugstetter Str. 55
D-79106 Freiburg i. Br.

Tel.: ++49-761-270 47560
Sekr.: ++49-761-270 47280
Fax: ++49-761-270 47390

benedikt.luka@uniklinik-freiburg.de
www.uniklinik-freiburg.de

Von: Jendrossek, Verena <Verena.Jendrossek@uk-essen.de>
Gesendet: Donnerstag, 25. November 2021 12:34
An: Lauber, Kirsten <Kirsten.Lauber@med.uni-muenchen.de>; ulrike.schoetz@staff.uni-marburg.de
Betreff: Re: CDDIS-21-2273R: Agreement to include Samet Mutlu into list of coauthors

Yes, I agree,
Verena

Von: Kristian Unger PD Dr. <unger@helmholtz-muenchen.de>
Gesendet: Donnerstag, 25. November 2021 12:38
An: Lauber, Kirsten <Kirsten.Lauber@med.uni-muenchen.de>
Cc: ulrike.schoetz@staff.uni-marburg.de; seyd.shnayien@gmail.com; Diana Klein <diana.klein@uk-essen.de>; steffen.spoerl@ukr.de; bertram.klinger@charite.de; "Dr. Julia Heß" <julia.hess@helmholtz-muenchen.de>; Orth, Michael Dr.rer.nat. <Michael.Orth@med.uni-muenchen.de>; Galabov, Radostin Dr.rer.nat. <Radostin.Galabov@med.uni-muenchen.de>; Hennel, Roman Dr.rer.nat. <Roman.Hennel@med.uni-muenchen.de>; benedikt.luka@uniklinik-freiburg.de; anja.sieber@charite.de; ute.ganswindt@i-med.ac.at; andreas.thomsen@uniklinik-freiburg.de; verena.jendrossek@uk-essen.de; Belka, Claus Prof.Dr. <Claus.Belka@med.uni-muenchen.de>; Nils Blüthgen <nils.bluethgen@charite.de>; steffen.unkel@med.uni-goettingen.de; Horst Zitzelsberger <zitzelsberger@helmholtz-muenchen.de>
Betreff: Re: CDDIS-21-2273R: Agreement to include Samet Mutlu into list of coauthors [signed OK]
Priorität: Hoch

Yes, I agree.
—
PD Dr. Kristian Unger
team leader Translational Bioinformatics and deputy head of research unit
Radiation Cytogenetics

Tel.: +49-89-3187-3515

Von: Hennel, Roman Dr.rer.nat. <Roman.Hennel@med.uni-muenchen.de>
Gesendet: Donnerstag, 25. November 2021 13:54
An: Lauber, Kirsten <Kirsten.Lauber@med.uni-muenchen.de>
Betreff: AW: CDDIS-21-2273R: Agreement to include Samet Mutlu into list of coauthors

Yes, agree.

Viele Grüße
Roman

Von: Julia Hess <julia.hess@helmholtz-muenchen.de>
Gesendet: Donnerstag, 25. November 2021 12:38
An: Lauber, Kirsten <Kirsten.Lauber@med.uni-muenchen.de>
Cc: ulrike.schoetz@staff.uni-marburg.de; seyd.shnayien@gmail.com; Diana Klein <diana.klein@uk-essen.de>; steffen.spoerl@ukr.de; Bertram Klinger <bertram.klinger@charite.de>; Orth, Michael Dr.rer.nat. <Michael.Orth@med.uni-muenchen.de>; Galabov, Radostin Dr.rer.nat. <Radostin.Galabov@med.uni-muenchen.de>; Hennel, Roman Dr.rer.nat. <Roman.Hennel@med.uni-muenchen.de>; benedikt.luka@uniklinik-freiburg.de; anja.sieber@charite.de; ute.ganswindt@i-med.ac.at; andreas.thomsen@uniklinik-freiburg.de; verena.jendrossek@uk-essen.de; Belka, Claus Prof.Dr. <Claus.Belka@med.uni-muenchen.de>; nils.bluethgen@charite.de; Kristian Unger <unger@helmholtz-muenchen.de>; steffen.unkel@med.uni-goettingen.de; Horst Zitzelsberger <zitzelsberger@helmholtz-muenchen.de>
Betreff: Re: CDDIS-21-2273R: Agreement to include Samet Mutlu into list of coauthors
Priorität: Hoch

Dear Kirsten,

Yes, agree.

BW Julia
Dr. Julia Heß-Rieger

Head of Therapeutic Targets and Intervention Group
Research Unit Radiation Cytogenetics

Phone: +49-89-3187-3517
Fax: +49-89-3187-2146

Von: Sieber, Anja <anja.sieber@charite.de>
Gesendet: Donnerstag, 25. November 2021 12:32
An: Lauber, Kirsten <Kirsten.Lauber@med.uni-muenchen.de>
Betreff: Re: [ext] CDDIS-21-2273R: Agreement to include Samet Mutlu into list of coauthors
Priorität: Hoch

Yes, agree.

Von: Blüthgen, Nils <nils.bluethgen@charite.de>
Gesendet: Donnerstag, 25. November 2021 12:32
An: Lauber, Kirsten <Kirsten.Lauber@med.uni-muenchen.de>
Cc: ulrike.schoetz@staff.uni-marburg.de; seyd.shnayien@gmail.com; diana.klein@uk-essen.de; steffen.spoerl@ukr.de; Klinger, Bertram <bertram.klinger@charite.de>; julia.hess@helmholtz-muenchen.de; Orth, Michael Dr.rer.nat. <Michael.Orth@med.uni-muenchen.de>; Galabov, Radostin Dr.rer.nat. <Radostin.Galabov@med.uni-muenchen.de>; Hennel, Roman Dr.rer.nat. <Roman.Hennel@med.uni-muenchen.de>; benedikt.luka@uniklinik-freiburg.de; Sieber, Anja <anja.sieber@charite.de>; ute.ganswindt@i-med.ac.at; andreas.thomsen@uniklinik-freiburg.de; verena.jendrossek@uk-essen.de; Belka, Claus Prof.Dr. <Claus.Belka@med.uni-muenchen.de>; unger@helmholtz-muenchen.de; steffen.unkel@med.uni-goettingen.de; zitzelsberger@helmholtz-muenchen.de
Betreff: Re: [ext] CDDIS-21-2273R: Agreement to include Samet Mutlu into list of coauthors

Yes, I agree

Von: Belka, Claus Prof.Dr. <Claus.Belka@med.uni-muenchen.de>
Gesendet: Donnerstag, 25. November 2021 12:31
An: Lauber, Kirsten <Kirsten.Lauber@med.uni-muenchen.de>
Betreff: AW: CDDIS-21-2273R: Agreement to include Samet Mutlu into list of coauthors

Yes, AGREE

Claus Belka

Von: Ute Ganswindt <ute.ganswindt@i-med.ac.at>
Gesendet: Donnerstag, 25. November 2021 12:29
An: Lauber, Kirsten <Kirsten.Lauber@med.uni-muenchen.de>
Betreff: AW: CDDIS-21-2273R: Agreement to include Samet Mutlu into list of coauthors

Yes, agree.

Univ.-Prof. Dr. med. Ute Ganswindt
Direktorin Univ.-Klinik für Strahlentherapie-Radioonkologie
Anichstr. 35 | 6020 Innsbruck | Tel +43 512 504 22800
Fax +43 512 504 22869
ute.ganswindt@i-med.ac.at
ute.ganswindt@tirol-kliniken.at

Von: Orth, Michael Dr.rer.nat. <Michael.Orth@med.uni-muenchen.de>
Gesendet: Donnerstag, 25. November 2021 12:28
An: Lauber, Kirsten <Kirsten.Lauber@med.uni-muenchen.de>
Cc: ulrike.schoetz@staff.uni-marburg.de; seyd.shnayien@gmail.com; diana.klein@uk-essen.de; steffen.spoerl@ukr.de; bertram.klinger@charite.de; julia.hess@helmholtz-muenchen.de; Galabov, Radostin Dr.rer.nat. <Radostin.Galabov@med.uni-muenchen.de>; Hennel, Roman Dr.rer.nat. <Roman.Hennel@med.uni-muenchen.de>; benedikt.luka@uniklinik-freiburg.de; anja.sieber@charite.de; ute.ganswindt@i-med.ac.at; andreas.thomsen@uniklinik-freiburg.de; verena.jendrossek@uk-essen.de; Belka, Claus Prof.Dr. <Claus.Belka@med.uni-muenchen.de>; nils.bluethgen@charite.de; unger@helmholtz-muenchen.de; steffen.unkel@med.uni-goettingen.de; zitzelsberger@helmholtz-muenchen.de
Betreff: AW: CDDIS-21-2273R: Agreement to include Samet Mutlu into list of coauthors

Yes, agree.

Von: Zitzelsberger, Horst, Prof. Dr. <zitzelsberger@helmholtz-muenchen.de>
Gesendet: Donnerstag, 25. November 2021 12:26
An: Lauber, Kirsten <Kirsten.Lauber@med.uni-muenchen.de>
Betreff: Re: CDDIS-21-2273R: Agreement to include Samet Mutlu into list of coauthors

Yes, agree!

Kind regards,

Horst Zitzelsberger

Prof. Dr. Horst Zitzelsberger
Abteilungsleiter/Head of Research Unit
Abteilung Strahlenzytogenetik/Research Unit Radiation Cytogenetics

Tel.: +49-89-31873421
Fax: +49-89-31872146
Email:Zitzelsberger@helmholtz-muenchen.de
